# Development of a Partial Proteolysis Targeting Chimera Library Based on Achiral Cereblon E3 Ligase Ligands and its Application for Bruton's Tyrosine Kinase Degraders

**DOI:** 10.1002/cmdc.202500209

**Published:** 2025-09-17

**Authors:** Chelsi M. Almodóvar‐Rivera, Ira Tandon, Ramesh Mudududdla, Paulina N. Esguerra, Kevin Lucio‐Acero, Weiping Tang

**Affiliations:** ^1^ Lachman Institute for Pharmaceutical Development School of Pharmacy University of Wisconsin‐Madison 777 Highland Avenue Madison WI 53705 USA; ^2^ Department of Chemistry University of Wisconsin‐Madison 1101 University Avenue Madison WI 53706 USA

**Keywords:** achiral ligands, Bruton's tyrosine kinase, cereblon, proteolysis targeting chimeras

## Abstract

Proteolysis targeting chimeras (PROTACs) offer a promising therapeutic approach by leveraging the ubiquitin–proteasome system (UPS) to degrade target proteins. These heterobifunctional molecules consist of a target protein ligand, an E3 ligase ligand, and a linker. Among the limited E3 ligase ligands available, cereblon (CRBN) ligands are the most widely used. However, the stability and racemization of current chiral CRBN ligands pose challenges for developing CRBN‐recruiting PROTAC therapeutics. Herein, a partial PROTAC library is reported incorporating an aldehyde motif and various linkers into previously developed achiral phenyl dihydrouracil CRBN ligands, which offer improved stability and eliminate racemization issues. This library enables the rapid generation of fully functional PROTACs targeting Bruton's tyrosine kinase (BTK) by coupling the aldehyde motif with a hydrazide‐containing BTK ligand. Initial HiBiT assay (Promega) screening identifies nine hits capable of significant BTK degradation, with compound B1 emerging as the most potent degrader. A stable amide bioisostere, AM‐B1, is further developed, which induces significant antiproliferation and BTK degradation. Mechanistic studies confirm BTK degradation via the UPS. This study highlights the development of an achiral CRBN‐based partial PROTAC library and demonstrates a two‐stage strategy for rapid PROTAC development against BTK.

## Graphical abstract

A partial proteolysis targeting chimera (PROTAC) library bearing achiral phenyl dihydrouracil cereblon ligands is prepared. It is then applied to the rapid development of a full PROTAC library targeting Bruton's tyrosine kinase, which can be directly used for screening.

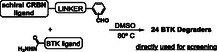

## 1 Introduction

Proteolysis targeting chimeras (PROTACs) have emerged as a promising therapeutic strategy for selectively degrading disease‐associated proteins of interest (POIs). These heterobifunctional molecules consist of a POI ligand, a linker, and an E3 ubiquitin ligase ligand.^[^ ^1^ ^]^ By bringing the POI and E3 ligase into close proximity, PROTACs promote POI polyubiquitination, leading to its subsequent proteasomal degradation. Since this process is catalytic, PROTACs can be recycled to induce multiple rounds of POI degradation.

**Figure 1:**
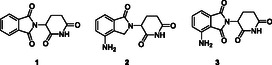
Structures of typical IMiD type of E3 CRBN ligands **1**) thalidomide, **2**) lenalidomide, and **3**) pomalidomide.^[^ ^14^ ^]^

Although more than 600 E3 ligases have been annotated in humans, only a few have been successfully employed in PROTACs. Among them, cereblon (CRBN) is the most commonly used E3 ligase in PROTACs due to the low molecular weight and drug‐like properties of its ligands, such as immunomodulatory drugs (IMiDs) including thalidomide, pomalidomide, lenalidomide, and their analogs (**Figure** **1**).^[^ ^1^ ^]^ However, thalidomide and its analogs can undergo rapid racemization and hydrolysis in vitro and in vivo.^[^ ^1^ ^,^ ^2^ ^]^ Different stereoisomers may exhibit distinct pharmacokinetic and pharmacodynamic profiles, including variations in toxicity and metabolism.^[^ ^3^ ^]^ These challenges complicate the therapeutic development of PROTACs using chiral CRBN ligands, highlighting the growing need for alternative CRBN binders for PROTAC development.^[^ ^1^ ^]^

**Figure 2:**
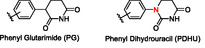
Alternative CRBN binders.

To date, various strategies have been developed to address the limitations of CRBN ligands, including replacing the hydrogen at the chiral carbon of thalidomide with deuterium or introducing a quaternary carbon.^[^ ^1^ ^]^ However, the addition of a methyl group significantly reduces the activity of thalidomide.^[^ ^1^ ^,^ ^4^ ^,^ ^5^ ^]^ Phenyl glutarimides (PGs) were recently developed as novel CRBN binders with improved hydrolytic stability and similar binding affinity compared to traditional IMiDs (**Figure** **2**).^[^ ^2^ ^]^ These ligands were incorporated into PG‐based bromodomain PROTACs, demonstrating potent degradation activity.^[^ ^2^ ^]^ Despite these advantages, PG ligands still contain a chiral center and, therefore, do not resolve the issue of racemization.

Our group recently reported a detailed structure–activity relationship (SAR) and stability studies of substituted and unsubstituted achiral phenyl dihydrouracil (PDHU) ligands as alternative CRBN binders (Figure 2).^[^ ^1^ ^]^ These ligands showed a similar binding affinity and greater stability than lenalidomide.^[^ ^1^ ^]^ In addition, five BRD4 PROTACs containing substituted PDHUs were also designed and synthesized, where significant degradation efficiency was observed in multiple cell lines.^[^ ^1^ ^]^ These findings suggest that PDHU ligands can be effectively applied in the design and synthesis of PROTACs for other POIs.

A significant challenge in PROTAC development is the extensive synthesis and screening required to optimize linker length, type, and position. To streamline this process, we previously introduced rapid‐TAC, a platform for the rapid synthesis of active PROTACs.^[^ ^6^ ^,^ ^7^ ^]^ Initially, a partial PROTAC library is generated by conjugating an aldehyde motif and various linkers to E3 ligase ligands. Fully functional PROTACs are then synthesized simply by mixing this library with a hydrazide‐containing POI ligand in DMSO. The aldehyde–hydrazide coupling reaction proceeds efficiently in DMSO with high conversion, generating water as the sole byproduct.^[^ ^6^ ^]^ This approach allows direct testing of the resulting PROTACs in cell‐based assays without requiring further purification.^[^ ^6^ ^]^ Additionally, the hydrolytically labile acyl‐hydrazone linker can be readily replaced with its isostere, such as an amide bond, to produce more drug‐like PROTACs.^[^ ^6^ ^]^

Building on our previous work, we herein report our efforts on the development of a partial PROTAC library by incorporating an aldehyde motif and various linkers into achiral PDHU‐based CRBN ligands, which offer improved stability and eliminate racemization concerns. This library enabled the rapid generation of fully functional PROTACs targeting Bruton's tyrosine kinase (BTK), a well‐validated therapeutic target implicated in various B‐cell malignancies, such as chronic lymphocytic leukemia (CLL), mantle cell lymphoma, diffuse large B‐cell lymphoma (DLBCL), and Waldenström macroglobulinemia.^[^ ^8^ ^,^ ^9^ ^]^ Recent studies indicate that inhibiting BTK's kinase activity alone is insufficient for effective treatment due to the role of its nonenzymatic domains in sustaining B‐cell receptor signaling, survival, and growth in CLL and DLBCL.^[^ ^10^ ^,^ ^11^ ^]^ These findings underscore the need for complete BTK elimination rather than mere kinase inhibition, further highlighting the therapeutic potential of BTK‐targeting PROTACs.^[^ ^10^ ^,^ ^11^ ^]^

## 2 Results and Discussion

### 2.1 Synthesis of Partial PROTAC Library for BTK Degraders

**Scheme 1:**
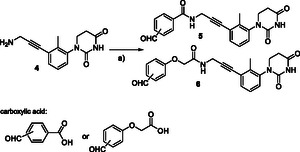
First set of partial PROTAC Library based on achiral CRBN E3 ligase ligands and alkyne linker. Reagents and conditions: a) HATU, DIPEA, DMF, rt, and carboxylic acid.

A series of partial PROTAC libraries based on the achiral PDHU ligand were synthesized starting with commercially available substituted anilines. Following our previous procedures,^[^ ^1^ ^]^ trisubstituted PDHU **4** with an amine functional group was prepared from the corresponding substituted aniline. Amide coupling reactions with the corresponding carboxylic acids bearing an aldehyde group led to the first set of six members with *ortho*, *meta*‐, and *para*‐substituted aldehyde for the achiral partial PROTAC library (**Scheme** **1**).

**Scheme 2:**
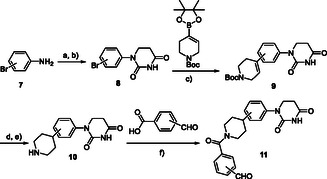
Second set of partial PROTAC library based on CRBN E3 ligase ligands and piperidine linker. Reagents and conditions: a) acrylic acid, toluene, 110 °C; b) urea, acetic acid, 120 °C; c) Pd(dppf)Cl_2_, KOAc, DMF, 100 °C; d) (i) H_2_/Pd/C, rt, e) TFA/DCM, rt; and f) HATU, DIPEA, DMF, rt.

We also developed another series of partial PROTAC library featuring a piperidine linker between the benzaldehyde and the achiral PDHU ligands (**Scheme** **2**). DPHUs **8** bearing a *meta*‐ and *para*‐substituted bromide were prepared via a condensation reaction. Next, a Suzuki coupling reaction provided the desired compounds **9**. Subsequently, hydrogenation followed by the deprotection of the Boc group afforded intermediate **10**. Finally, an amide coupling reaction was performed, resulting in another series of six members in the achiral partial PROTAC library, incorporating *ortho*, *meta*‐, and *para*‐substituted aldehydes.

**Scheme 3:**
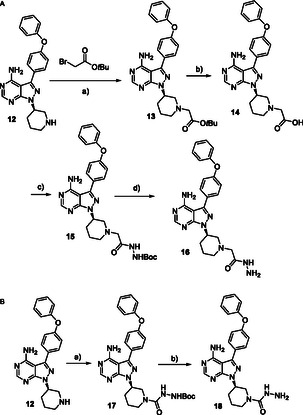
Synthesis of BTK ligands bearing a hydrazide or semicarbazide. Reagents and conditions: Scheme A: a) K_2_CO_3_; b) TFA, DCM, rt, 4 h; c) NH_2_NHBoc, HATU, DIPEA, DMF, rt; and d) TFA, DCM, rt, 1 h; Scheme B: a) NH_2_NHBoc, CDI, THF, rt; b) TFA, DCM, rt, 1 h.

The synthesis of the BTK binders was designed to follow the synthetic route outlined in **Scheme** **3**, starting from a commercially available ibrutinib precursor, which contains a free secondary amine in a piperidine moiety. We selected two functional groups that readily react with aldehyde—hydrazide and semicarbazide (Scheme 3). The synthesis of the first binder, shown in Scheme 3A, involved an alkylation reaction, hydrolysis, amide formation, and Boc deprotection, ultimately yielding the desired hydrazide **16**. Similarly, the synthesis of the second BTK binder, shown in Scheme 3B, was carried out through a one‐pot reaction utilizing a Boc‐protected hydrazine and CDI at room temperature. The reaction proceeded through the formation of a Boc‐protected semicarbazide, which was then deprotected to afford the final semicarbazide **18**.

### 2.2 Synthesis and Biological Screening of BTK Degraders

**Figure 3:**
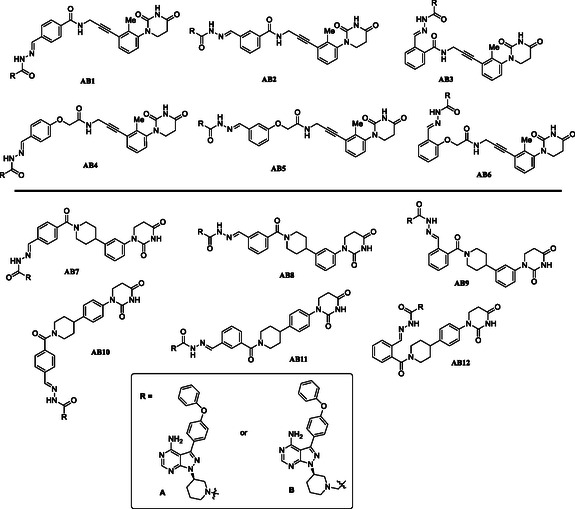
BTK degraders based on achiral CRBN ligands.

With the partial PROTAC library and BTK warhead building blocks in hand, fully functional PROTACs were synthesized by mixing the partial PROTAC library with the hydrazide‐ or semicarbazide‐containing BTK ligands in DMSO following our previously established procedures.^[^ ^6^ ^]^ Specifically, we created the achiral CRBN ligand‐based BTK degraders composed of two building blocks containing a BTK binding ligand and a hydrazide or semicarbazide‐containing achiral CRBN ligands, which differ from each other by the length and position of the linkers (**Figure** **3**). To test the activity of this library of compounds, we employed a BTK‐HiBiT assay (Promega) developed in Ramos cells and evaluated compounds at two different concentrations (10, 1 μM) for 8 h.

**Figure 4:**
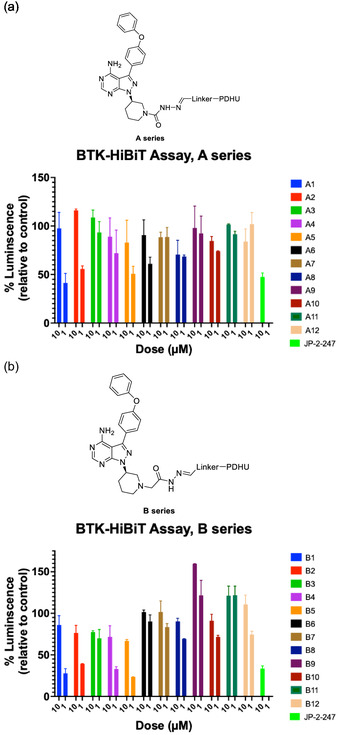
Screening of BTK degraders based on achiral PDHU CRBN ligands in BTK‐HiBiT assay. Relative luminescence of degrader‐treated cells in BTK‐HiBiT assay; a) A‐series compounds are based on semicarbazide linkage; b) B‐series compounds are based on hydrazide linkage.

Our results showed a significant loss of luminescence signals for multiple compounds, indicating the degradation of BTK. In some cases, the degradation appeared to be on par with the positive control (BTK degrader JP‐2‐247)^[^ ^12^ ^]^ (**Figure** **4**). Our initial BTK‐HiBiT screening suggests that significant degradation was achieved by employing our previously reported achiral CRBN E3 ligase ligand^[^ ^1^ ^]^ with *meta* or *para* linker on the phenyl group in the linker region.

Moreover, while these results also demonstrated that significant BTK degradation was achieved by degraders containing either hydrazide or semicarbazide, compounds with an additional methylene group in the hydrazide exhibited slightly better activity (Figure 4). This improvement may be attributed to increased linker flexibility, potentially facilitating the formation of a more stable ternary complex. Additionally, these findings highlight that even small differences in linker length and orientation can significantly impact the degradation efficiency. Based on these results, we chose compound B1 as the most promising hit for further structural modifications, as it achieved the most significant degradation at 1 μM (Figure 4B). Notably, compound A1 from the A series and B1 from the B series demonstrated substantial degradation, distinguishing them from other compounds evaluated in their respective series.

### 2.3 AM‐B1 Dose Response, Antiproliferation, and Time Course

**Figure 5:**
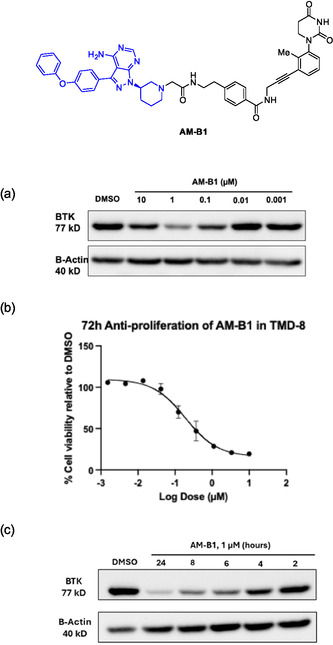
Cellular assays confirm that AM‐B1 is a potent BTK degrader in TMD‐8 cells. a) Response of AM‐B1 at indicated concentrations for 8 h. b) Antiproliferation assay after 72 h. c) Time course analyses of AM‐B1 at indicated timepoints at 1 µM.

Since the acylhydrazone motif is known to be hydrolytic labile, we next replaced the acylhydrazone motif in BTK degrader B1 (Figure 3) by its amide bioisostere to improve the stability.^[^ ^6^ ^]^ The stable BTK PROTAC AM‐B1 was prepared, and a dose response study was conducted for this degrader in TMD‐8 cells to probe the effective concentration range (**Figure** **5**a). Not surprisingly, AM‐B1 showed significant BTK degradation starting at 0.1 μM and optimal degradation at 1 μM at 8 h with a DC_50_ around 0.014 μM. A “hook effect” was also noticeable at 10 μM (Figure 5A). We also tested AM‐B1′s ability to induce growth inhibition in TMD‐8 cells (Figure 5B). Consistent with AM‐B1's ability to induce BTK degradation, the cell proliferation showed an effective antiproliferation and half‐maximal inhibition concentration (IC_50_) of 0.19 μM (Figure 5b). Moreover, a time course study was conducted to determine the kinetics. Treatment of TMD‐8 cells with 1 μM of AM‐B1 showed that BTK degradation could be achieved starting at 4 h and maximum degradation was obtained at 24 h (Figure 5c).

### 2.4 AM‐B1 Mechanism and Competition Assay

**Figure 6:**
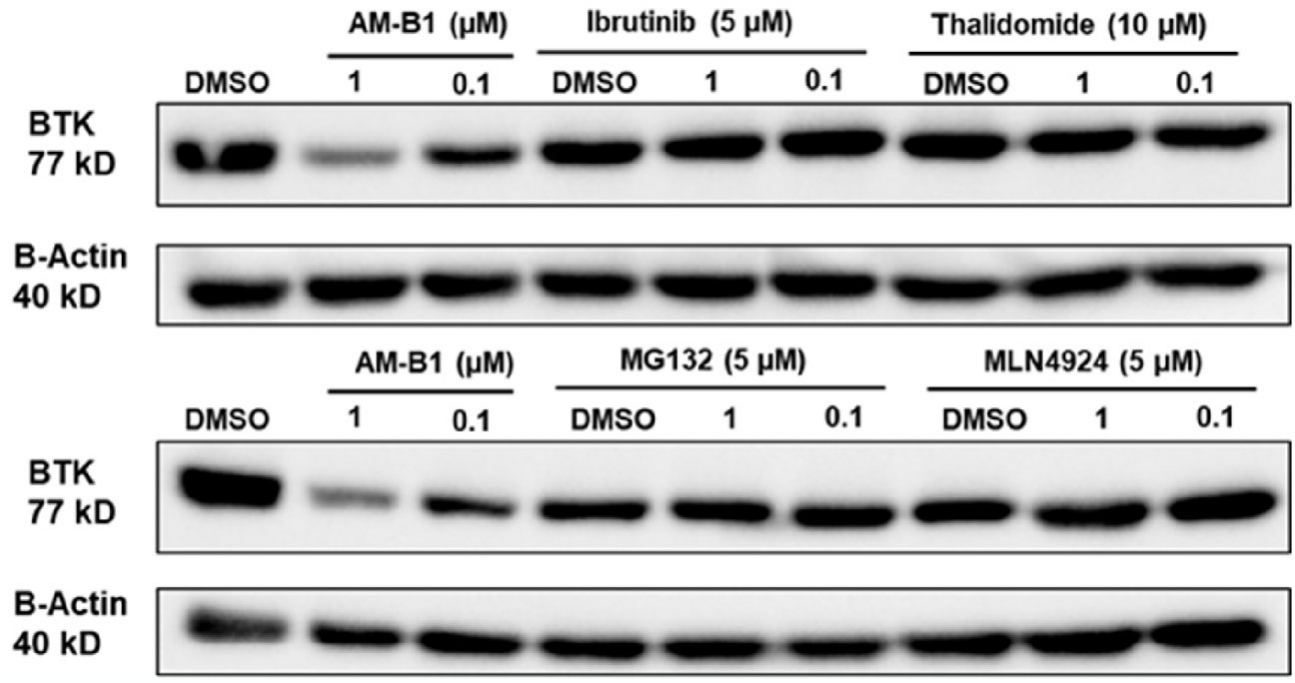
Mechanistic studies to confirm the activity of AM‐B1.

To validate that AM‐B1 induces BTK degradation through the ubiquitin‐proteasome system (UPS), TMD‐8 cells were cotreated with different pathway competitors. Cells were pretreated with the CRBN ligand thalidomide (10 μM) or BTK ligand ibrutinib (5 μM) for 2 h, followed by coincubation of either 1 or 0.1 μM of AM‐B1 for an additional 6 h. The presence of the CRBN ligand and BTK ligands induced a complete rescue of the BTK protein levels (**Figure** **6**). Additionally, the pretreatment with either the proteasome inhibitor MG132 (5 μM) or neddylation inhibitor MLN4924 (5 μM) for 2 h, followed by coincubation of 1 or 0.1 μM of AM‐B1 for 6 h, also showed a significant abolishment of BTK degradation (Figure 6). These results indicate that AM‐B1 degrades BTK through ubiquitination and proteasomal degradation.

## 3 Conclusion

In this study, we described the synthesis of a partial PROTAC library based on achiral PDHU ligands and the application of a two‐stage strategy for the rapid development of BTK degraders. The initial library consisted of twelve PDHU‐based CRBN ligands featuring relatively short and rigid linkers with variations in *ortho*, *meta*, or *para* phenyl substitutions. These subtle structural modifications allowed for a systematic examination of the effects of linker length and orientation over a relatively short distance. We further demonstrated the utility of this partial PROTAC library in the development of BTK degraders. Our initial BTK‐HiBiT assay identified nine active degraders. The SAR confirms that PROTAC linker orientation plays a significant role in degradation efficiency, particularly for compounds with short linkers. Notably, linkers containing *para*‐ and *meta*‐substituted phenyl groups attached to an alkyne‐based linker exhibited the highest BTK degradation. Based on these findings, we selected compound B1 for further studies and designed its stable analog AM‐B1, bearing an amide isostere. Dose‐response and time‐course experiments indicated that AM‐B1 achieved optimal BTK degradation at 1 μM within 24 h, with a DC_50_ in the low micromolar range. Mechanistic studies confirmed that BTK degradation proceeded via the UPS, consistent with traditional PROTACs.

Thus, this study demonstrated the feasibility of using a two‐stage strategy for the rapid identification of BTK‐targeting PROTACs. Moreover, we identified AM‐B1 as a promising BTK degrader featuring an achiral PDHU‐CRBN ligand, which has the potential to overcome limitations associated with chiral CRBN ligands, such as rapid racemization and stability issues.^[^ ^13^ ^]^

## Supporting Information

The authors have cited additional references within the Supporting Information.^[^ ^14^ ^]^

## Conflict of Interest

W.T. is a shareholder in Chimergen Therapeutics Inc., NovaCure Biosciences Holding Inc., and MolTenon Biosciences Inc. W.T. also receives research funding from Novo Nordisk and MolTenon Biosciences Inc.

## Supporting information

Supplementary Material

## Data Availability

The data that support the findings of this study are available in the supplementary material of this article.

## References

[1] H. Xie , C. Li , H. Tang , I. Tandon , J. Liao , B. L. Roberts , Y. Zhao , W. Tang , J. Med. Chem. 2023, 66, 2904. 36749666 10.1021/acs.jmedchem.2c01941 PMC10398712

[2] J. Min , A. Mayasundari , F. Keramatnia , B. Jonchere , S. W. Yang , J. Jarusiewicz , M. Actis , S. Das , B. Young , J. Slavish , L. Yang , Y. Li , X. Fu , S. H. Garrett , M. Yun , Z. Li , S. Nithianantham , S. Chai , T. Chen , A. Shelat , R. E. Lee , G. Nishiguchi , S. W. White , M. F. Roussel , P. R. Potts , M. Fischer , Z. Rankovic , Angew. Chem., Int. Ed. 2021, 60, 26663. 10.1002/anie.202108848 PMC8648984 34614283

[3] M. M. Coelho , C. Fernandes , F. Remião , M. E. Tiritan , Molecules 2021, 26, 3113. 34070985 10.3390/molecules26113113 PMC8197169

[4] K. Nishimura , Y. Hashimoto , S. Iwasaki , Chem. Pharm. Bull. 1994, 42, 1157. 10.1248/cpb.42.1157 8069968

[5] J. D. Hansen , M. Correa , M. A. Nagy , M. Alexander , V. Plantevin , V. Grant , B. Whitefield , D. Huang , T. Kercher , R. Harris , R. K. Narla , J. Leisten , Y. Tang , M. Moghaddam , K. Ebinger , J. Piccotti , C. G. Havens , B. Cathers , J. Carmichael , T. Daniel , R. Vessey , L. G. Hamann , K. Leftheris , D. Mendy , F. Baculi , L. A. LeBrun , G. Khambatta , A. Lopez‐Girona , J. Med. Chem. 2020, 63, 6648. 32130004 10.1021/acs.jmedchem.9b01928

[6] B. L. Roberts , Z.‐X. Ma , A. Gao , E. D. Leisten , D. Yin , W. Xu , W. Tang , ACS Chem. Biol. 2020, 15, 1487. 32255606 10.1021/acschembio.0c00140

[7] L. Guo , J. Liu , X. Nie , T. Wang , Z. Ma , D. Yin , W. Tang , Bioorg. Med. Chem. Lett. 2022, 75, 128982. 36096343 10.1016/j.bmcl.2022.128982

[8] S. Chen , Z. Chen , L. Lu , Y. Zhao , R. Zhou , Q. Xie , Y. Shu , J. Lin , X. Yu , Y. Wang , Eur. J. Med. Chem. 2023, 255, 115403. 37119666 10.1016/j.ejmech.2023.115403

[9] S. Pal Singh , F. Dammeijer , R. W. Hendriks , Mol. Cancer 2018, 17, 57. 29455639 10.1186/s12943-018-0779-z PMC5817726

[10] S. Montoya , J. Bourcier , M. Noviski , H. Lu , M. C. Thompson , A. Chirino , J. Jahn , A. K. Sondhi , S. Gajewski , Y. S. Tan , S. Yung , A. Urban , E. Wang , C. Han , X. Mi , W. J. Kim , Q. Sievers , P. Auger , H. Bousquet , N. Brathaban , B. Bravo , M. Gessner , C. Guiducci , J. N. Iuliano , T. Kane , R. Mukerji , P. J. Reddy , J. Powers , M. Sanchez Garcia De Los Rios , J. Ye , et al., Science 2024, 383, eadi5798. 38301010 10.1126/science.adi5798 PMC11103405

[11] H. Yuan , Y. Zhu , Y. Cheng , J. Hou , F. Jin , M. Li , W. Jia , Z. Cheng , H. Xing , M. Liu , T. Han , J. Biol. Chem. 2022, 298, 102555. 36183831 10.1016/j.jbc.2022.102555 PMC9636578

[12] E. S. Toriki , J. W. Papatzimas , K. Nishikawa , D. Dovala , A. O. Frank , M. J. Hesse , D. Dankova , J.‐G. Song , M. Bruce‐Smythe , H. Struble , F. J. Garcia , S. M. Brittain , A. C. Kile , L. M. McGregor , J. M. McKenna , J. A. Tallarico , M. Schirle , D. K. Nomura , ACS Cent. Sci. 2023, 9, 915. 37252349 10.1021/acscentsci.2c01317 PMC10214506

[13] C. M. Almodóvar‐Rivera , Z. Zhang , J. Li , H. Xie , Y. Zhao , L. Guo , M. G. Mannhardt , W. C. Tang , ChemBioChem 2023, 24, e202300482. 37418320 10.1002/cbic.202300482 PMC10591699

[14] P. P. Chamberlain , A. Lopez‐Girona , K. Miller , G. Carmel , B. Pagarigan , B. Chie‐Leon , E. Rychak , L. G. Corral , Y. J. Ren , M. Wang , M. Riley , S. L. Delker , T. Ito , H. Ando , T. Mori , Y. Hirano , H. Handa , T. Hakoshima , T. O. Daniel , B. E. Cathers , Nat. Struct. Mol. Biol. 2014, 21, 803. 25108355 10.1038/nsmb.2874

