# Supplementary material for: Development of a Partial Proteolysis Targeting Chimera Library Based on Achiral Cereblon E3 Ligase Ligands and its Application for Bruton's Tyrosine Kinase Degraders
Source: ChemMedChem. 2025 Sep 17;20(22):e202500209. doi: 10.1002/cmdc.202500209 (PMC12471865; doi:10.1002/cmdc.202500209)

Supporting Information

Development of a Partial PROTAC Library Based on Achiral Cereblon E3 Ligase Ligands and Its Application for Bruton's Tyrosine Kinase Degraders

Chelsi M. Almodóvar-Rivera,[a]# Ira Tandon,[a]# Ramesh Mudududdla,[a]# Paulina N. Esguerra,[a] Kevin Lucio-Acero, and Weiping Tang*[a][b]

C. M. Almodóvar-Rivera, I. Tandon, R. Mudududdla, P. N. Esguerra, K. Lucio-Acero, W. Tang
Lachman Institute for Pharmaceutical Development
School of Pharmacy, University of Wisconsin-Madison
777 Highland Avenue, Madison, WI 53705 (USA)
Email: weiping.tang@wisc.edu

[b] W. Tang
Department of Chemistry
University of Wisconsin-Madison
1101 University Avenue, Madison, WI 53706 (USA)

# These authors contributed equally.

1. Biological Procedures

1.1. Cell Culture

The Ramos BTK-HiBiT cell line was generously gifted by Promega. TMD-8 cell line was generously gifted by the Lixin Rui Lab at the University of Wisconsin Madison. All cells were cultured in RPMI-1640 media (Corning) supplemented with 10% fetal bovine serum (FBS), 1% Sodium Pyruvate, 1% Penicillin/Streptomycin, and 10 mM HEPES at 37℃ under a humidified 5% CO_2 atmosphere.

1.2. HiBiT Screening Assay

Ramos BTK-HiBiT cells were seeded at 50,000 cells/well in a volume of 100 µL. The cells were then treated with the indicated dose of compounds at no greater than 0.5% DMSO. Plates were incubated at 37℃ under a humidified 5% CO_2 atmosphere for indicated time point. Luminescence was measured using the Nano-Glo HiBiT Lytic Detection System (#N3040, Promega). Prior to reading, cells and buffer were equilibrated to room temperature. 50 µL of cell volume was transferred to a white-wall 96-well plate along with 50 µL of prepared buffer and substrate mix. Samples were prepared according to provided protocol and luminescence was detected on a plate reader (BMG CLARIOstar Plus).

1.3. Western Blotting

Cells were treated with the indicated concentrations of compounds under the indicated time. Cells were washed with cold PBS twice, then lysed with RIPA buffer for 15 minutes on ice. The supernatant was collected after centrifugation at 16,000 × g at 4 ℃ for 15 minutes. The protein concentration was determined using BCA (Bicinchoninic Acid) assay. 10 µg total protein was loaded and then separated by SDS-PAGE. Protein was transferred to polyvinylidene fluoride (PVDF) membranes, then blocked with 5% non-fat milk, and probed by following primary antibodies: BTK (D3H5, #8547, 1:1000, CST), pMAPK (E10, #9106, 1:1000, CST), pSTAT3 (D3A7 XP, #9145, 1:1000, CST), and β-Actin (C4, #sc-47778, 1:1000, Santa Cruz. The membrane was washed with 1X TBS-T 3 times for 5 minutes and incubated with Anti-rabbit IgG (#7074, 1:5000, CST) and Anti-mouse IgG (#7076, 1:5000, CST) HRP-linked secondary antibodies for 1 hour at room temperature. The membrane was incubated with ECL substrates (#170-5061, Bio-rad) for 5 minutes and visualized by using a Bio-Rad Chemi-Doc MP imaging system. Band intensities were quantified by BioRad Image Lab software.

1.4. Cell Viability Assay

Cells were seeded at varying concentrations in a 96-well cell culture plate at a density of $2x10^4$ cells/well for suspension cells in 100 µL media. The cells were then treated with the indicated dose of compounds at no greater than 0.5% DMSO. Plates were incubated at 37℃ under a humidified 5% CO_2 atmosphere for 72 h. 10 µL of 10X Alamar Blue stock (0.5mg/mL) was added into each well and incubated for 1-4h. The fluorescence was measured using a 560 nm excitation/590 nm emission filter set in a plate reader (BMG

Omega). Relative cell viabilities in each well were calculated by normalizing the read value to the value of DMSO-treated wells. Growth curves were generated by plotting relative fluorescence units vs. compound concentration and graphed using GraphPad Prism software (version 10.3.1).

2. Experimental Procedures

2.1. General

All solvents and reagents were purchased from commercially available sources. Thin-layer chromatography (TLC) was used with precoated silica gel plates. Flash column chromatography was performed using silica gel. The ^{1}H and ^{13}C nuclear magnetic resonance (NMR) spectra were recorded using a Bruker AV-400 MHz in parts per million (ppm) (δ) downfield of TMS (δ = 0). Signal splitting patterns were described as singlet (s), doublet (d), triplet (t) or multiplet (m), with coupling constants (*J*) in hertz. The liquid chromatography–mass spectrometry (LC–MS) analysis of final products was processed on an Agilent 1290 Infinity II LC system using a Poroshell 120 EC-C18 column (5 cm × 2.1 mm, 1.9 μm) for chromatographic separation. Agilent 6120 Quadrupole LC/MS with multimode electrospray ionization plus atmospheric pressure chemical ionization was used for detection. The mobile phases were 5.0% methanol and 0.1% formic acid in purified water (A) and 0.1% formic acid in methanol (B). The gradient was held at 5% (0–0.2 min), increased to 100% at 2.5 min, then held at isocratic 100% B for 0.4 min, and then immediately stepped back down to 5% for 0.1 min re-equilibration. The flow rate was set at 0.8 mL/min. The column temperature was set at 40 °C. High resolution mass spectra (HRMS) were performed by Analytical Instrument Center at the School of Pharmacy or Department of Chemistry on an Electron Spray Injection (ESI) mass spectrometer.

2.2. General procedure for dihydropyrimidine-2,4(1H,3H)-diones (8a-c).

A solution of halogenated aniline (**7a-c**) (1.75g, 10.17 mmol) and toluene (22 mL) was prepared. Acrylic acid (22.7 mmol) was then added to this solution, which was stirred at a temperature of 110 °C for 5 hours. After this, the toluene was concentrated under reduced pressure. Next, acetic acid (26 mL) and urea (52.2 mmol) were added to the mixture. The reaction was stirred at 120 °C for 16 hours. Following this period, the acetic acid was evaporated using a rotary evaporator. The resulting products (**8a-c**) were then used directly for the next step.

*1-(3-bromophenyl) dihydropyrimidine-2,4(1H,3H)-dione **(8a)***

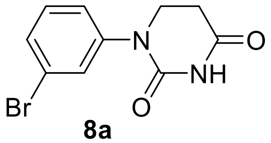

8a

^{1}H NMR (400 MHz, DMSO) δ 10.41 (s, 1H), 7.56 (t, *J* = 1.9 Hz, 1H), 7.43 (dt, *J* = 7.2, 1.9 Hz, 1H), 7.42 – 7.29 (m, 2H), 3.81 – 3.74 (m, 2H), 2.70 (t, *J* = 6.7 Hz, 2H); ^{13}C NMR (101 MHz, DMSO) δ 171.32, 152.58, 143.80, 131.06, 129.14, 128.63, 124.62, 121.47, 44.87, 31.31; LCMS (*m/z*): 270.1 $[M+H]^+$

1-(4-bromophenyl) dihydropyrimidine-2,4(1H,3H)-dione ***(8b)***

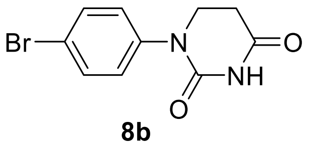

8b

^{1}H NMR (400 MHz, DMSO) δ 10.43 (s, 1H), 7.62 – 7.54 (m, 2H), 7.35 – 7.27 (m, 2H), 3.79 (t, *J* = 6.7 Hz, 2H), 2.71 (t, *J* = 6.7 Hz, 2H); ^{13}C NMR (101 MHz, DMSO) δ 171.01, 152.51, 141.79, 131.91, 127.75, 118.56, 44.75, 31.44; LCMS (*m/z*): 270.1 $[M+H]^+$

1-(3-iodo-2-methylphenyl)dihydropyrimidine-2,4(1H,3H)-dione ***(8c)***

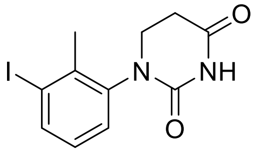

8c

^{1}H NMR (400 MHz, DMSO-d_6) δ 7.82 (dd, *J* = 7.9, 1.2 Hz, 1H), 7.33 (dd, *J* = 7.9, 1.2 Hz, 1H), 7.08 – 6.98 (m, 1H), 3.78 (ddd, *J* = 12.2, 9.8, 5.2 Hz, 1H), 3.52 (dt, *J* = 12.1, 6.0 Hz, 1H), 2.80 (ddd, *J* = 16.1, 9.8, 6.1 Hz, 1H), 2.67 (dt, *J* = 16.7, 5.5 Hz, 1H), 2.28 (s, 3H); LCMS (*m/z*): 331.1 $[M+H]^+$

2.3. Synthesis of compound 9a-9b using Suzuki conditions

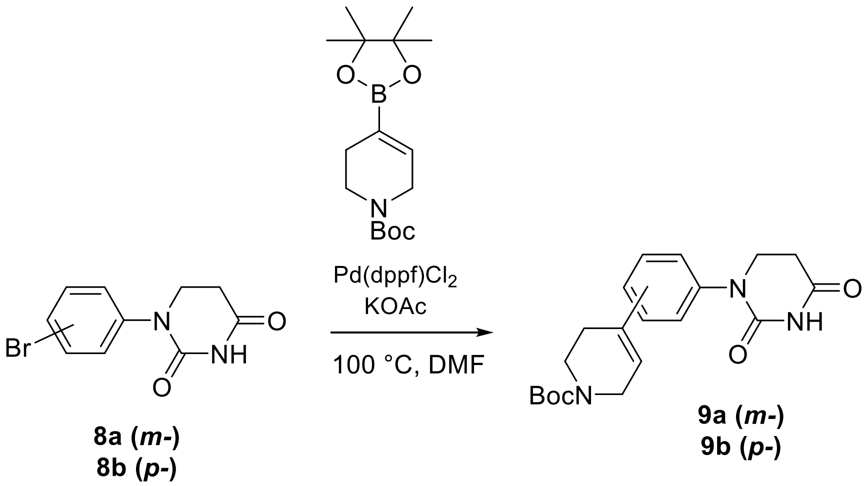

8a (*m*-)
8b (*p*-)

9a (*m*-)
9b (*p*-)

As previously described[14] a solution of **8a** or **8b** (100 mg, 0.371 mmol) in DMF (1.8 mL, 0.2 M) was added the corresponding boron pinacol ester or boronic acid compounds (0.540 mmol), Pd(dppf)Cl_2 (13.6 mg, 0.0186 mmol) and KOAc (109.4 mg, 1.1 mmol) at room temperature. Then, the reaction mixture was stirred for 12 h at 100 °C. Afterwards, the solvent was concentrated by rotovapor and the sample was diluted with DCM. The crude was later purified by flash column chromatography using an elution gradient of 0-10% MeOH in DCM as mobile phase and a 12 g silica column as stationary phase. Finally, the desired fractions were combined and concentrated to afford the desired compounds (**9a, b**).

*tert-butyl 4-(3-(2,4-dioxotetrahydropyrimidin-1(2H)-yl)phenyl)-3,6-dihydropyridine-1(2H)-carboxylate (**9a**)*

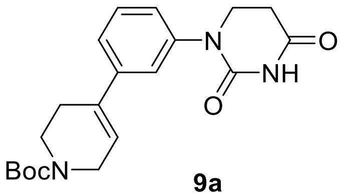

9a

^{1}H NMR (400 MHz, $CDCl_3$) δ 7.56 (s, 1H), 7.31 (t, *J* = 7.8 Hz, 1H), 7.26 – 7.18 (m, 2H), 7.15 – 7.07 (m, 1H), 5.98 (s, 1H), 4.00 (d, *J* = 3.6 Hz, 2H), 3.81 (t, *J* = 6.6 Hz, 2H), 3.56 (t, *J* = 5.7 Hz, 2H), 2.77 (t, *J* = 6.7 Hz, 2H), 2.44 (s, 2H), 1.42 (s, 9H); ^{13}C NMR (101 MHz, $CDCl_3$) δ 169.43, 151.79, 142.25, 141.35, 129.42, 123.84, 123.76, 122.11, 79.92, 77.47, 77.15, 76.84, 75.18, 45.44, 31.57, 28.63, 25.00, 24.70; LCMS (*m/z*): 372.4 $[M+H]^+$

*tert-butyl 4-(4-(2,4-dioxotetrahydropyrimidin-1(2H)-yl)phenyl)-3,6-dihydropyridine-1(2H)-carboxylate **(9b)***

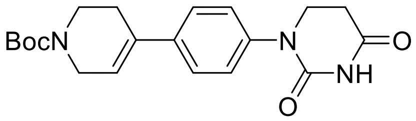

9b

^{1}H NMR (400 MHz, $CDCl_3$) δ 7.53 (s, 1H), 7.42 – 7.28 (m, 2H), 7.27 – 7.06 (m, 3H), 5.97 (s, 1H), 4.03 (d, *J* = 13.9 Hz, 2H), 3.81 (t, *J* = 6.7 Hz, 2H), 3.57 (t, *J* = 5.7 Hz, 2H), 2.77 (t, *J* = 6.7 Hz, 2H), 1.42 (s, 9H); ^{13}C NMR (101 MHz, $CDCl_3$) δ 169.37, 155.08, 152.89, 151.73, 140.06, 139.55, 136.41, 125.89, 124.99, 83.56, 79.90, 79.58, 45.30, 31.54, 28.64, 26.33; LCMS (*m/z*): 372.4 $[M+H]^+$

2.4. Synthesis of partial PROTAC library aldehydes (11aa-ac, 11ba-bc):

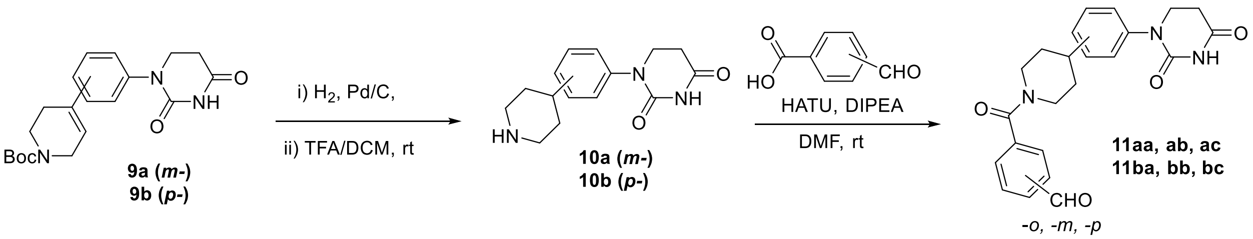

A solution containing **9a** or **9b** (262 mg, 0.705 mmol) in methanol (40 mL) was added 10% Pd/C (89 mg). The reaction was purged with argon and hydrogen. The solution was stirred at room temperature for 48 hours. Afterward, the palladium was removed by filtration, and the reaction mixture was concentrated under vacuum and used directly in the next step. To a solution of **9aa** or **9ba** (1.0 eq) in DCM was added TFA (1.5 mL) at 0 °C. The reaction mixture was stirred at room temperature for 1 h. Finally, the solvent and TFA were evaporated in vacuo to give the crude products (**10a, b**) which were directly used in the next step.To a solution of the benzaldehyde-COOH (1.0 eq) in DMF was added HATU (1.0 eq), DIPEA (2.0 eq) and the deprotected crude of the amine (**10a** or **b**). The reaction mixture was stirred at rt for 4 h. The mixture was then extracted with ethyl acetate (3 x 10 mL). The combined organic fractions were dried over Na_2SO_4 and concentrated. The crude was later purified by flash column chromatography (elution gradient of 0-10% MeOH in DCM). The desired fractions were combined and concentrated to yield our desired compounds (**11aa-ac; 11ba-bc**).

*4-(4-(3-(2,4-dioxotetrahydropyrimidin-1(2H)-yl)phenyl)piperidine-1-carbonyl)benzaldehyde **(11aa)***

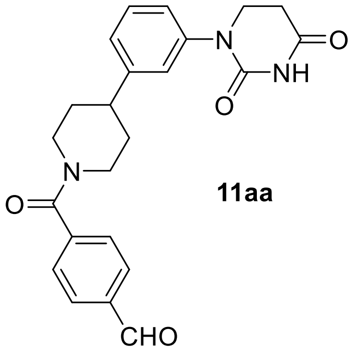

11aa

^{1}H NMR (400 MHz, DMSO) δ 10.28 (s, 1H), 9.98 (d, *J* = 8.5 Hz, 1H), 8.32 (s, 2H), 8.11 (d, *J* = 7.6 Hz, 1H), 7.95 – 7.87 (m, 2H), 7.81 (d, *J* = 7.6 Hz, 1H), 7.71 (d, *J* = 7.6 Hz, 1H), 7.62 (t, *J* = 7.6 Hz, 1H), 7.47 (t, *J* = 7.6 Hz, 1H), 7.30 – 7.17 (m, 2H), 7.14 – 7.07 (m, 2H), 3.72 (t, *J* = 6.6 Hz, 2H), 2.63 (t, *J* = 6.7 Hz, 2H), 1.92 (s, 1H), 1.61 (s, 3H) (missing aliphatic peaks are hiding under DMSO water peak); LCMS (*m/z*): 406.2 $[M+H]^+$

*3-(4-(3-(2,4-dioxotetrahydropyrimidin-1(2H)-yl)phenyl)piperidine-1-carbonyl)benzaldehyde **(11ab)***

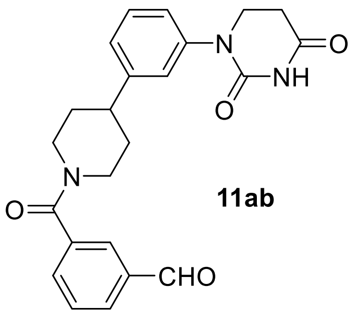

11ab

^{1}H NMR (400 MHz, DMSO-d_6) δ 10.35 (s, 1H), 10.05 (d, J = 8.5 Hz, 1H), 8.39 (s, 1H), 8.06 – 7.91 (m, 2H), 7.83 – 7.64 (m, 2H), 7.36 – 7.08 (m, 3H), 4.65 (s, 1H), 3.78 (d, J = 13.2 Hz, 2H), 2.96 – 2.77 (m, 3H), 2.70 (t, J = 6.7 Hz, 3H), 1.89 (s, 1H), 1.67 (s, 3H); LCMS (*m/z*): 406.2 $[M+H]^+$

2-(4-(3-(2,4-dioxotetrahydropyrimidin-1(2H)-yl)phenyl)piperidine-1-carbonyl)benzaldehyde ***(11ac)***

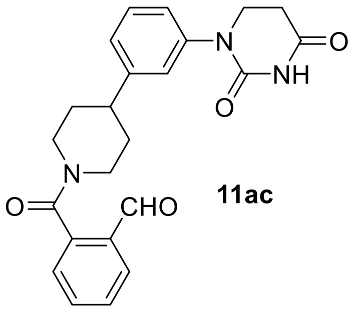

11ac

^{1}H NMR (400 MHz, DMSO) δ 10.35 (s, 1H), 10.03 (s, 1H), 7.83 (dq, J = 21.0, 10.1 Hz, 2H), 7.71 – 7.51 (m, 5H), 7.36 – 7.21 (m, 2H), 7.16 (t, J = 8.6 Hz, 2H), 4.71 (d, J = 13.3 Hz, 1H), 3.78 (s, 1H), 3.54 (s, 2H), 2.74 – 2.66 (m, 2H), 1.99 (s, 1H), 1.90 (d, J = 12.7 Hz, 1H), 1.68 (dd, J = 24.3, 12.6 Hz, 2H) (missing peaks are hiding under DMSO-d_6); ^{13}C NMR (101 MHz, DMSO) δ 192.21, 171.11, 160.64, 152.73, 142.62, 134.84, 132.84, 131.60, 131.10, 129.79, 127.47, 125.35, 124.18, 114.34, 107.83, 97.50, 69.77, 56.90, 47.50, 45.13, 33.15, 31.56, 23.89; LCMS (*m/z*): 406.2 $[M+H]^+$

4-(4-(4-(2,4-dioxotetrahydropyrimidin-1(2H)-yl)phenyl)piperidine-1-carbonyl)benzaldehyde ***(11ba)***

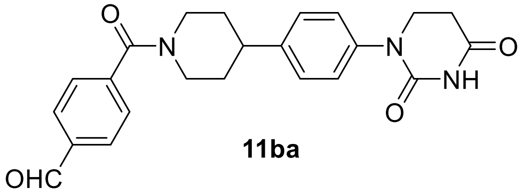

11ba

^{1}H NMR (400 MHz, DMSO-*d*6) δ 10.35 (s, 1H), 10.05 (d, J = 7.8 Hz, 1H), 8.32 (s, 1H), 8.11 – 7.82 (m, 3H), 7.82 – 7.52 (m, 2H), 7.39 – 7.10 (m, 2H), 4.64 (s, 1H), 3.83 – 3.73 (m, 2H), 3.28 – 3.08 (m, 3H), 2.86 (d, J = 13.0 Hz, 2H), 2.69 (td, J = 6.7, 3.5 Hz, 2H), 1.88 (s, 1H), 1.67 (s, 2H); LCMS (*m/z*): 406.2 $[M+H]^+$

3-(4-(4-(2,4-dioxotetrahydropyrimidin-1(2H)-yl)phenyl)piperidine-1-carbonyl)benzaldehyde ***(11bb)***

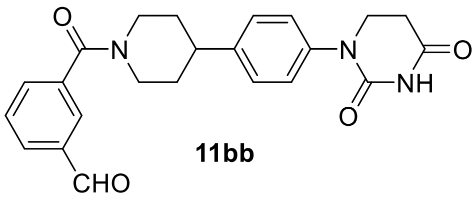

11bb

^{1}H NMR (400 MHz, DMSO-d_6) δ 10.39 – 10.31 (m, 1H), 10.04 (d, *J* = 7.4 Hz, 1H), 8.07 – 7.88 (m, 4H), 7.38 – 7.12 (m, 4H), 4.74 – 4.61 (m, 1H), 3.83 – 3.74 (m, 3H), 3.08 (dd, *J* = 15.0, 10.8 Hz, 2H), 2.99 – 2.77 (m, 2H), 2.77 – 2.62 (m, 2H), 1.95 – 1.81 (m, 1H), 1.79 – 1.59 (m, 2H); LCMS (*m/z*): 406.2 $[M+H]^+$

2-(4-(4-(2,4-dioxotetrahydropyrimidin-1(2H)-yl)phenyl)piperidine-1-carbonyl)benzaldehyde ***(11bc)***

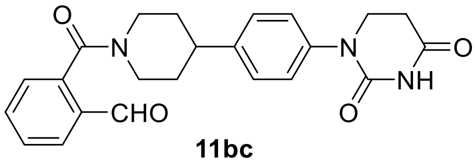

11bc

^{1}H NMR (400 MHz, DMSO-d_6) δ 10.03 (s, 1H), 7.99 (d, *J* = 7.3 Hz, 1H), 7.81 – 7.74 (m, 1H), 7.67 (d, *J* = 7.4 Hz, 1H), 7.63 – 7.48 (m, 2H), 7.48 – 7.40 (m, 1H), 7.40 – 7.33 (m, 1H), 7.33 – 7.21 (m, 2H), 4.73 (d, *J* = 8.7 Hz, 1H), 3.78 (dt, *J* = 15.9, 6.8 Hz, 2H), 3.20 – 3.05 (m, 2H), 2.96 – 2.77 (m, 2H), 2.74 – 2.61 (m, 2H), 2.34 (dd, *J* = 3.7, 1.9 Hz, 1H), 1.96 – 1.85 (m, 1H), 1.74 – 1.55 (m, 2H); LCMS (*m/z*): 406.2 $[M+H]^+$

2.5. *Synthesis of 1-(3-(3-aminoprop-1-yn-1-yl)-2-methylphenyl)dihydropyrimidine-2,4(1H,3H)-dione hydrochloride (4)*

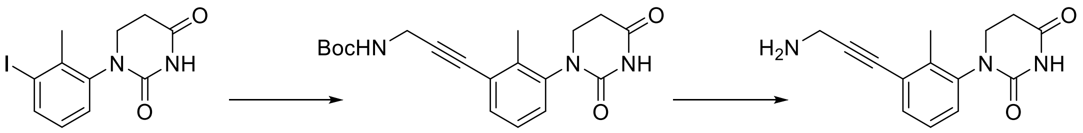

8c **Boc-4** **4**

Tert-butyl (3-(3-(2,4-dioxotetrahydropyrimidin-1(2H)-yl)-2-methylphenyl)prop-2-yn-1-yl)carbamate (***Boc-4***)*:*[1]

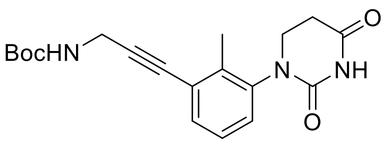

Boc-4

The compound **Boc-4** was synthesized as previously described using **8c**.[1] A 25 mL flask was charged with a magnetic stirring bar, compound **8c** (350 mg, 1.06 mmol), N-Boc-propargylamine(493 mg, 3.18 mmol), $Pd(PPh_3)_2Cl_2$ (37.2 mg, 0.053 mmol), and CuI (10.1 mg, 0.053 mmol), evacuated, and backfilled with argon. Dimethylformamide (MF) (2.5 mL) and triethylamine (NEt_3) (2.5 mL) were successively added using a syringe. The reaction mixture was stirred at room temperature until the starting material compound 4disappeared, as indicated by TLC. The mixture was partitioned between ethyl acetate and a saturated solution of sodium bicarbonate; the organic layer was washed with brine, dried over Na_2SO_4, and concentrated under vacuum. The residue was purified by column chromatography on silica to provide **Boc-4** as a white solid. ^{1}H NMR (400 MHz, Chloroform-*d*) δ 7.50 (s, 1H), 7.41 (dd, *J* = 7.5, 1.5 Hz, 1H), 7.25 – 7.10 (m, 2H), 4.18 (d, *J* = 5.6 Hz, 2H), 3.79 (ddd, *J* = 12.7, 8.8, 5.9 Hz, 1H), 3.62 (dt, *J* = 12.5, 6.1 Hz, 1H), 2.88 – 2.79 (m, 2H), 2.34 (s, 3H), 1.47 (s, 9H); LCMS (*m/z*): 358.1 $[M + H]^+$

*1-(3-(3-aminoprop-1-yn-1-yl)-2-methylphenyl)dihydropyrimidine-2,4(1H,3H)-dione hydrochloride (**4**):*

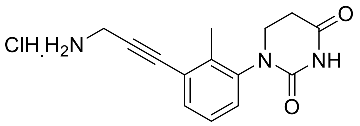

4

To a stirring solution of tert-butyl (3-(3-(2,4-dioxotetrahydropyrimidin-1(2H)-yl)-2-methylphenyl)prop-2-yn-1-yl)carbamate (**Boc-4**) (0.38g, 1.06 mmol) in DCM (1 ml) was added 4 M HCl in dioxane (1.33 ml, 5.32 mmol) and the resulting mixture was allowed to stir at room temperature for 30 minutes. After completion of reaction, all the volatiles were removed on rotavap, resulting solid was washed with diethyl ether and dried to get the title compound (0.263 g, 96 % yield). ^{1}H NMR (400 MHz, DMSO-d_6) δ 10.39 (s, 1H), 8.48 (bs, 3H; NH_3^+), 7.39 (ddd, *J* = 13.5, 7.8, 1.4 Hz, 2H), 7.29 (t, *J* = 7.7 Hz, 1H), 4.04 (s, 2H), 3.79 (ddd, *J* = 12.2, 9.8, 5.2 Hz, 1H), 3.51 (dt, *J* = 12.0, 5.9 Hz, 1H), 2.81 (ddd, *J* = 16.1, 9.8, 6.1 Hz, 1H), 2.68 (dt, *J* = 16.7, 5.5 Hz, 1H), 2.30 (s, 3H); LCMS (*m/z*): 258.1 $[M + H]^+$

2.6. General procedure for synthesis of aldehyde precursors 5a-c and 6a-c

To a solution of the benzaldehyde-COOH (1.0 eq) in DMF was added HATU (1.0 eq), DIPEA (2.0 eq), and the propargyl amine (**4**). The reaction mixture was stirred at rt for 4 h. The mixture was then extracted with ethyl acetate (3 x 10 mL). The combined organic fractions were dried over Na_2SO_4 and concentrated. The crude was later purified by flash column chromatography (elution gradient of 0-10% MeOH in DCM). The desired fractions were combined and concentrated to yield our desired compounds (**5a-c; 6a-c**).

*N-(3-(3-(2,4-dioxotetrahydropyrimidin-1(2H)-yl)-2-methylphenyl)prop-2-yn-1-yl)-4-formylbenzamide **(5a)***

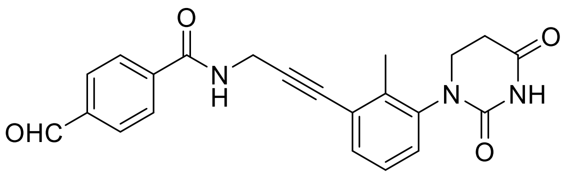

5a

^{1}H NMR (400 MHz, DMSO) δ 10.38 (s, 1H), 10.10 (s, 1H), 9.33 (t, *J* = 5.3 Hz, 1H), 8.11 – 7.99 (m, 4H), 7.35 (ddd, *J* = 29.5, 7.8, 1.5 Hz, 2H), 7.28 – 7.18 (m, 1H), 4.43 – 4.37 (m, 2H), 3.77 (ddd, *J* = 12.1, 9.8, 5.3 Hz, 1H), 3.51 (dt, *J* = 12.0, 5.9 Hz, 1H), 2.79 (ddd, *J* = 18.8, 9.7, 6.1 Hz, 1H), 2.73 – 2.62 (m, 1H), 2.26 (d, *J* = 7.9 Hz, 3H); LCMS (*m/z*): 390.1 $[M+H]^+$

N-(3-(3-(2,4-dioxotetrahydropyrimidin-1(2H)-yl)-2-methylphenyl)prop-2-yn-1-yl)-3-formylbenzamide ***(5b)***

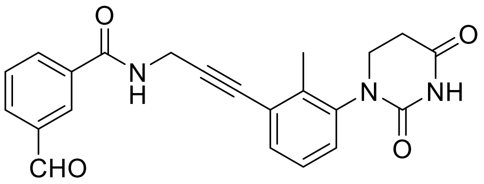

5b

^{1}H NMR (400 MHz, DMSO) δ 10.38 (s, 1H), 9.88 (s, 1H), 7.88 (d, *J* = 8.7 Hz, 2H), 7.40 – 7.20 (m, 4H), 7.16 (d, *J* = 8.7 Hz, 2H), 4.24 (d, *J* = 5.4 Hz, 2H), 3.80 (d, *J* = 13.9 Hz, 1H), 3.52 – 3.47 (m, 1H), 2.82 – 2.73 (m, 1H), 2.72 – 2.65 (m, 2H), 2.34 (m, 1H), 2.27 (s, 1H); LCMS (*m/z*): 390.1 $[M+H]^+$

N-(3-(3-(2,4-dioxotetrahydropyrimidin-1(2H)-yl)-2-methylphenyl)prop-2-yn-1-yl)-2-formylbenzamide ***(5c)***

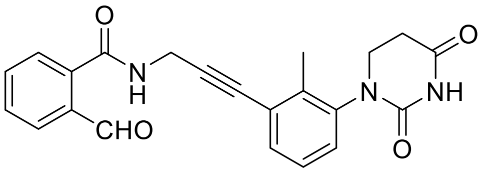

5c

^{1}H NMR (400 MHz, DMSO-d_6) δ 10.35 (d, *J* = 5.1 Hz, 1H), 8.36 – 8.05 (m, 1H), 8.04 – 7.46 (m, 6H), 7.40 – 7.10 (m, 2H), 3.78 (m, 2H), 2.90 (q, *J* = 7.1 Hz, 3H), 2.71 (dd, *J* = 12.3, 5.7 Hz, 2H), 2.30 (s, 1H), 2.19 – 2.06 (m, 1H); LCMS (*m/z*): 390.1 $[M+H]^+$

N-(3-(3-(2,4-dioxotetrahydropyrimidin-1(2H)-yl)-2-methylphenyl)prop-2-yn-1-yl)-2-(4-formylphenoxy)acetamide ***(6a)***

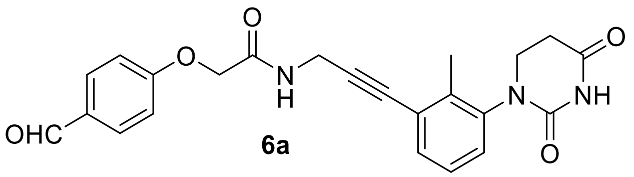

6a

^{1}H NMR (400 MHz, DMSO) δ 10.38 (s, 1H), 9.88 (s, 1H), 8.82 (t, *J* = 5.6 Hz, 1H), 7.91 – 7.84 (m, 2H), 7.33 (ddd, *J* = 15.5, 7.8, 1.6 Hz, 2H), 7.24 (t, *J* = 7.7 Hz, 1H), 7.16 (d, *J* = 8.6 Hz, 2H), 4.71 (s, 2H), 4.24 (d, *J* = 5.6 Hz, 2H), 3.83 – 3.69 (m, 1H), 3.51 (dt, *J* = 12.0, 5.9 Hz, 1H), 2.87 – 2.75 (m, 1H), 2.70 (d, *J* = 5.8 Hz, 1H), 2.24 (s, 3H); ^{13}C NMR (101 MHz, DMSO) δ 191.41, 170.75, 167.09, 162.56, 151.79, 141.30, 137.79, 131.74, 130.16, 127.79, 126.74, 123.41, 115.25, 115.07, 91.05, 80.09, 66.89, 44.48, 31.08, 28.67, 15.75; LCMS (*m/z*): 420.2 $[M+H]^+$

N-(3-(3-(2,4-dioxotetrahydropyrimidin-1(2H)-yl)-2-methylphenyl)prop-2-yn-1-yl)-2-(3-formylphenoxy)acetamide ***(6b)***

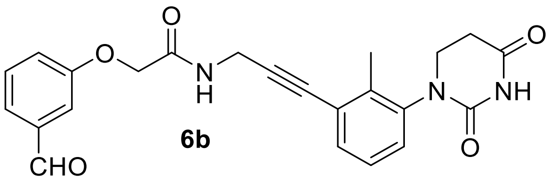

6b

^{1}H NMR (400 MHz, DMSO) δ 10.37 (s, 1H), 9.97 (s, 1H), 8.77 (t, *J* = 5.7 Hz, 1H), 7.56 (d, *J* = 4.8 Hz, 2H), 7.47 (d, *J* = 2.6 Hz, 1H), 7.33 (dd, *J* = 15.2, 7.5 Hz, 3H), 7.24 (t, *J* = 7.7 Hz, 1H), 4.66 (s, 2H), 4.24 (d, *J* = 5.6 Hz, 2H), 3.83 – 3.69 (m, 1H), 3.51 (dt, *J* = 12.0, 5.9 Hz, 1H), 2.84 – 2.77 (m, 2H), 2.23 (s, 3H); ^{13}C NMR (101 MHz, DMSO) δ 193.31, 171.19, 167.82, 158.67, 152.23, 141.75, 138.05, 130.90, 128.25, 127.18, 123.75, 121.91, 114.44, 91.56, 80.50, 67.38, 52.40, 47.79, 44.94, 37.45, 34.00, 31.55, 29.39. LCMS (*m/z*): 420.2 $[M+H]^+$

N-(3-(3-(2,4-dioxotetrahydropyrimidin-1(2H)-yl)-2-methylphenyl)prop-2-yn-1-yl)-2-(2-formylphenoxy)acetamide ***(6c)***

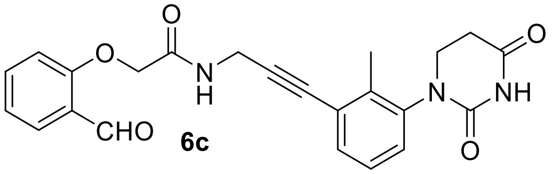

6c

^{1}H NMR (400 MHz, DMSO) δ 10.51 (s, 1H), 10.38 (s, 1H), 8.76 (t, *J* = 5.7 Hz, 1H), 7.75 (dd, *J* = 7.6, 1.8 Hz, 1H), 7.68 – 7.59 (m, 1H), 7.33 (ddd, *J* = 16.1, 7.7, 1.6 Hz, 2H), 7.25 (t, *J* = 7.7 Hz, 1H), 7.19 – 7.09 (m, 2H), 4.76 (s, 2H), 4.26 (d, *J* = 5.6 Hz, 2H), 3.83 – 3.70 (m, 1H), 3.51 (dt, *J* = 12.0, 5.9 Hz, 1H), 2.81 (dd, *J* = 16.6, 6.2 Hz, 1H), 2.79 – 2.68 (m, 1H), 2.24 (s, 3H); ^{13}C NMR (101 MHz, DMSO) δ 190.00, 170.75, 167.21, 159.75, 151.79, 141.30, 137.80, 130.82, 128.96, 127.93, 127.78, 126.75, 123.41, 121.44, 113.57, 113.56, 91.08, 80.06, 67.21, 44.48, 31.09, 28.69, 15.73; LCMS (*m/z*): 420.2 $[M+H]^+$

2.7. Synthesis of achiral dihydrouracil aldehyde library for partial PROTAC synthesis

To a solution of the benzaldehyde-COOH (1.0 eq) in DMF was added HATU (1.0 eq), DIPEA (2.0 eq) and the deprotected crude of the amine. The reaction mixture was stirred at rt for 4 h. The mixture was then extracted with ethyl acetate (3 x 10 mL). The combined organic fractions were dried over Na_2SO_4 and concentrated. The crude was later purified by flash column chromatography (elution gradient of 0-10% MeOH in DCM). The desired fractions were combined and concentrated to yield our desired compounds.

2.8. Synthesis of BTK ligands based on noncovalent linkers.

Synthesis of tert-butyl (R)-2-(3-(4-amino-3-(4-phenoxyphenyl)-1H-pyrazolo[3,4-d]pyrimidin-1-yl)piperidin-1-yl)acetate ***(14-INT-a)***

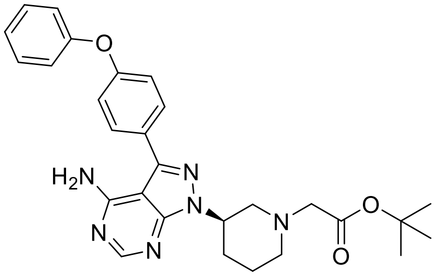

14-INT-a

To a solution of *S*)-3-(4-phenoxyphenyl)-1-(piperidin-3-yl)-1*H*-pyrazolo[3,4-*d*]pyrimidin-4-amine (300 mg, 0.776 mmol) in DMF (3.5 mL), 2-(*tert*-butoxy)acetyl bromide (226 mg, 1.16 mmol) and Cs_2CO_3 (214 g, 1.55 mmol) were added. The solution was stirred at 60°C overnight. The reaction mixture was then cooled at room temperature and an extraction was done using H_2O and ethyl acetate (3 x 50 mL). The organic layer was separated, dried with Na_2SO_4 and concentrated. The crudes were later purified using flash column chromatography DCM/MeOH 0-10% to achieve the desired product. ^{1}H NMR (400 MHz, DMSO-d_6) δ 8.24 (s, 1H), 7.72 – 7.59 (m, 2H), 7.49 – 7.38 (m, 2H), 7.24 – 7.07 (m, 5H), 4.77 (tt, *J* = 10.4, 4.8 Hz, 1H), 3.20 (s, 2H), 3.01 (dd, *J* = 10.4, 4.1 Hz, 1H), 2.84 (d, *J* = 11.0 Hz, 1H), 2.76 (t, *J* = 10.6 Hz, 1H), 2.33 – 2.22 (m, 1H), 1.98 (td, *J* = 9.6, 3.7 Hz, 2H), 1.85 – 1.74 (m, 1H), 1.74 – 1.58 (m, 1H), 1.39 (s, 9H); LCMS (*m/z*): 501.1 $[M + H]^+$

Synthesis of (R)-2-(3-(4-amino-3-(4-phenoxyphenyl)-1H-pyrazolo[3,4-d]pyrimidin-1-yl)piperidin-1-yl)acetic acid ***(14)***

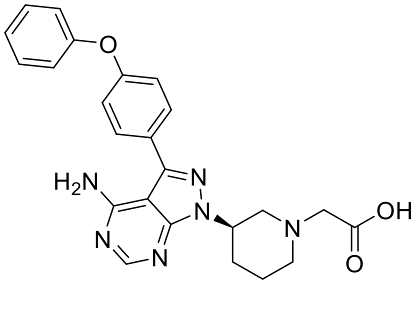

14

To a solution of **14 INT-a** (1.0 eq) in 2 mL of DCM was added 2 mL of TFA at 0 °C, and the resulting reaction mixture was slowly warmed to room temperature and stirred for 1 h. Once the reaction was complete, as indicated by TLC or LCMS, all excess volatiles were evaporated on a rotavap to get the crude product. The crude product was triturated with diethyl ether, resulting solid which was directly used in the next step. ^{1}H NMR (400 MHz, DMSO-d_6) δ 8.50 – 8.29 (m, 1H), 7.67 (d, J = 8.3 Hz, 2H), 7.53 – 7.37 (m, 2H), 7.28 – 6.98 (m, 5H), 5.28 (d, J = 9.3 Hz, 1H), 4.22 (d, J = 2.2 Hz, 3H), 3.77 – 3.47 (m, 2H), 3.33 – 3.09 (m, 1H), 2.22 – 1.90 (m, 4H); LCMS (*m/z*): 445.1 $[M + H]^+$

Synthesis of (S)-2-(3-(4-amino-3-(4-phenoxyphenyl)-1H-pyrazolo[3,4-d] pyrimidin-1-yl)piperidin-1-yl)acetohydrazide **(16)**

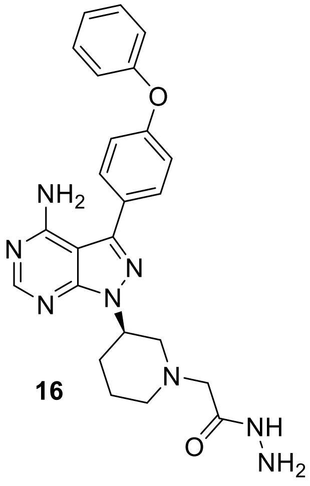

16

To a solution of the (*S*)-2-(3-(4-amino-3-(4-phenoxyphenyl)-1*H*-pyrazolo[3,4-*d*] pyrimidin-1-yl) piperidin-1-yl)acetic acid **14** (1.0 eq) in DMF was added HATU (1.0 eq), DIPEA (2.0 eq), and 2.0 eq of tert-butyl carbamate. The reaction mixture was stirred at rt for 4 h. The mixture was then extracted with ethyl acetate (3 x 10 mL). The combined organic fractions were dried over Na_2SO_4 and concentrated. The crude was later purified by flash column chromatography (elution gradient of 0-10% MeOH in DCM). The desired fractions were combined and concentrated to yield our desired compound (**15**). After the compound was

purified, 1.0 eq was dissolved in DCM and TFA (1:1 mixture, 4 mL) at 0 °C. The reaction mixture was stirred at room temperature for 1 h. The DCM and TFA were evaporated in vacuo, and the resulting crude product was triturated with diethyl ether to get the desired product (**16**). ^{1}H NMR (400 MHz, DMSO-d_6) δ 9.47 (s, 1H), 8.69 (s, 1H), 8.15 (s, 1H), 7.67 (d, *J* = 8.6 Hz, 2H), 7.53 – 7.34 (m, 2H), 7.29 – 7.04 (m, 5H), 5.56 – 5.36 (m, 1H), 4.27 (s, 2H), 3.77 (d, *J* = 7.6 Hz, 2H), 3.66 – 3.55 (m, 1H), 3.31 (d, *J* = 12.4 Hz, 1H), 2.30 – 1.93 (m, 4H); ^{13}C NMR (101 MHz, DMSO) δ 163.02, 158.00, 156.02, 152.85, 151.69, 147.76, 146.77, 130.32, 130.25, 125.71, 124.08, 119.20, 119.08, 96.98, 66.40, 54.57, 53.48, 53.23, 51.81, 50.32; LCMS (*m/z*): 459.2 $[M+H]^+$

Synthesis of (S)-3-(4-amino-3-(4-phenoxyphenyl)-1H-pyrazolo[3,4-d] pyrimidin-1-yl) piperidine-1-carbohydrazide trifluoro acetic acid salt ***(18)***

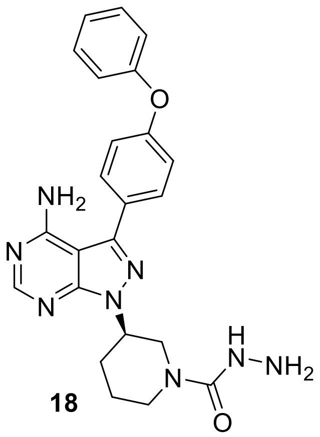

18

To a round bottom flask, NH_2NHBoc (2 g, 15.13 mmol) and CDI (2.5 g, 15.13 mmol) were dissolved in THF (65 mL). The solution was stirred at room temperature for 2 h. Afterwards, (*S*)-3-(4-phenoxyphenyl)-1-(piperidin-3-yl)-1*H*-pyrazolo[3,4-*d*] pyrimidin-4-amine (2.9 g, 7.55 mmol) was directly added to the reaction mixture. The reaction was stirred at room temperature for 24 h. Finally, the THF was concentrated in vacuo and the crude was later purified by flash column chromatography (elution gradient of 0-10% MeOH in DCM). Then, 1.0 eq was dissolved in DCM and TFA (1:1 mixture, 4 mL) at 0 °C. The reaction mixture was stirred at room temperature for 1 h. The DCM and TFA were evaporated in vacuo to give the crude product, which was triturated with diethyl ether to get the desired product.^{1}H NMR (400 MHz, DMSO-d_6) δ 9.83 – 9.71 (m, 1H), 9.13 (t, *J* = 1.3 Hz, 1H), 8.57 (d, *J* = 3.6 Hz, 1H), 7.71 – 7.64 (m, 4H), 7.48 – 7.41 (m, 2H), 7.23 – 7.10 (m, 4H), 4.75 (tt, *J* = 10.4, 4.4 Hz, 1H), 4.25 – 4.17 (m, 1H), 3.96 (d, *J* = 13.3 Hz, 1H), 3.38 (dd, *J* = 12.8, 10.7 Hz, 1H), 3.09 – 2.92 (m, 1H), 2.37 – 2.04 (m, 2H), 2.03 – 1.61 (m, 2H); LCMS (*m/z*): 445.2 $[M+H]^+$

2.9. General procedure for the preparation of BTK PROTACs under miniaturized conditions

Solution **A**: A 50 mM DMSO stock solution of the BTK building blocks was prepared by dissolving appropriate amounts in DMSO.

Solution **B**: A 50 mM DMSO stock solution of each member of the aldehyde achiral partial PROTAC library was also prepared similarly to Solution **A**.

We next mixed 20 µL of DMSO solution **A** with 20 µL of DMSO solution **B** in a 96-well plate. The resulting mixture was then heated at 80 °C for 3-4 h on the reaction block. After cooling it to room temperature, another 60 µL of DMSO was added to the solution. The solution was then cooled down to room temperature and the purity of the product was analyzed by LC-MS. All products were directly used for cell-based screening without any further manipulations.

3. Synthesis of stable BTK PROTAC AM-B1

*Synthesis of tert-butyl (4-((3-(3-(2,4-dioxotetrahydropyrimidin-1(2H)-yl)-2-methylphenyl)prop-2-yn-1-yl)carbamoyl)phenethyl)carbamate **(19)***

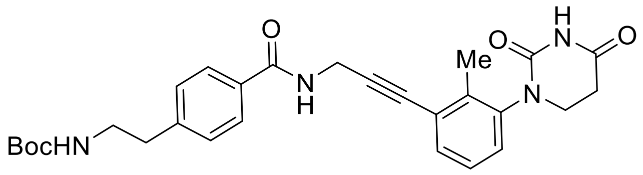

19

To a solution of the 4-(2-((tert-butoxycarbonyl)amino)ethyl)benzoic acid (0.25g, 0.942 mmol), 1-(3-(3-aminoprop-1-yn-1-yl)-2-methylphenyl)dihydropyrimidine-2,4(1H,3H)-dione hydrochloride (**4**) (0.276 g, 0.942 mmol), in DMF was added HATU (0.716 g, 1.88 mmol), DIPEA (0.4925 ml, 2.83 mmol). The reaction mixture was stirred at room temperature for 4 h. The mixture was then extracted with ethyl acetate (3 x 50 mL). The combined organic fractions were dried over Na_2SO_4 and concentrated. The crude was purified by flash column chromatography (elution gradient of 0-10% MeOH in DCM). The desired fractions were combined and concentrated to yield the title compound. ^{1}H NMR (400 MHz, DMSO-d_6) δ 10.36 (s, 1H), 8.97 (t, *J* = 5.5 Hz, 1H), 7.86 – 7.75 (m, 2H), 7.36 (dd, *J* = 7.5, 1.5 Hz, 1H), 7.32 – 7.19 (m, 4H), 6.89 (t, *J* = 5.6 Hz, 1H), 4.35 (d, *J* = 5.5 Hz, 2H), 3.76 (ddd, *J* = 12.2, 9.8, 5.2 Hz, 1H), 3.49 (dt, *J* = 12.1, 5.9 Hz, 1H), 3.21 – 3.06 (m, 2H), 2.85 – 2.60 (m, 4H), 2.25 (s, 3H), 1.36 (s, 9H); LCMS (*m/z*): 505.2 $[M + H]^+$

*Synthesis of 4-(2-aminoethyl)-N-(3-(3-(2,4-dioxotetrahydropyrimidin-1(2H)-yl)-2-methylphenyl)prop-2-yn-1-yl)benzamide hydrochloride salt **(20)***

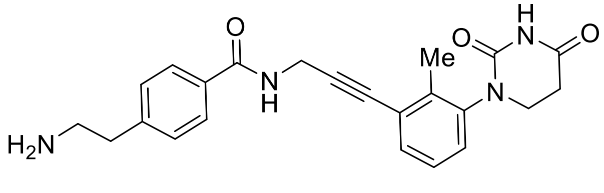

20

To a stirring solution of *tert*-butyl (4-((3-(3-(2,4-dioxotetrahydropyrimidin-1(2H)-yl)-2-methylphenyl)prop-2-yn-1-yl)carbamoyl)phenethyl)carbamate (**19**) (0.3 g, 0.594 mmol) in DCM (1 ml) was added 4 M HCl in dioxane (1.49 ml, 5.95 mmol) and the resulting mixture was allowed to stir at room temperature for 30 minutes. After completion of reaction, all the volatiles were removed on rotavap, resulting solid was washed with diethyl ether and dried to get the title compound (0.256 g, 97 % yield). ^{1}H NMR (400 MHz, DMSO-d_6) δ 10.35 (bs, 1H), 9.02 (t, *J* = 5.6 Hz, 1H), 8.39 (s, 1H), 7.84 (d, *J* = 8.1 Hz, 2H), 7.41 – 7.32 (m, 3H), 7.30 (dd, *J* = 7.9, 1.5 Hz, 1H), 7.23 (t, *J* = 7.7 Hz, 1H), 4.35 (d, *J* = 5.5 Hz, 2H), 3.76 (ddd, *J* = 12.3, 9.8, 5.2 Hz, 1H), 3.49 (dt, *J* = 12.1, 5.9 Hz, 1H), 2.96 (t, *J* = 7.6 Hz, 2H), 2.90 – 2.72 (m, 3H), 2.66 (dt, *J* = 16.6, 5.5 Hz, 1H), 2.25 (s, 3H); LCMS (*m/z*): 405.1 $[M + H]^+$

(R)-4-(2-(2-(3-(4-amino-3-(4-phenoxyphenyl)-1H-pyrazolo[3,4-d]pyrimidin-1-yl)piperidin-1-yl)acetamido)ethyl)-N-(3-(3-(2,4-dioxotetrahydropyrimidin-1(2H)-yl)-2-methylphenyl)prop-2-yn-1-yl)benzamide ***(AM-B1)***

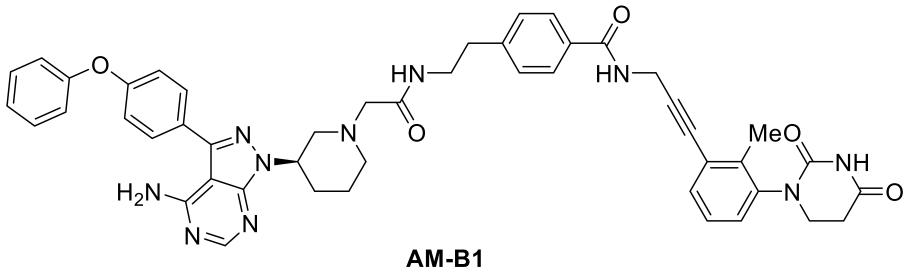

AM-B1

To a solution of the (R)-2-(3-(4-amino-3-(4-phenoxyphenyl)-1H-pyrazolo[3,4-d]pyrimidin-1-yl)piperidin-1-yl)acetic acid (**14**) (86 mg, 0.193 mmol), 4-(2-aminoethyl)-N-(3-(3-(2,4-dioxotetrahydropyrimidin-1(2H)-yl)-2-methylphenyl)prop-2-yn-1-yl)benzamide hydrochloride (**20**) (85.3 mg, 0.193 mmol) in DMF (2 ml) was added HATU (110.3 mg, 0.290 mmol), DIPEA (101.1 µL, 0.580 mmol). The reaction mixture was stirred at room temperature for 4 h. The mixture was then extracted with ethyl acetate (3 x 10 mL). The combined organic fractions were dried over Na_2SO_4 and concentrated. The crude was later purified by flash column chromatography (elution gradient of 0-10% MeOH in DCM) and followed by prep HPLC to yield the desired compound (42 mg, 26% yield, purity 99% at 254 nM). ^{1}H NMR (400 MHz, Methanol-d_4) δ 8.28 (s, 1H), 8.16 (s, 2H), 7.77 – 7.59 (m, 4H), 7.37 (dddd, *J* = 12.8, 7.1, 5.8, 1.9 Hz, 3H), 7.28 (dd, *J* = 8.1, 3.3 Hz, 2H), 7.24 – 7.10 (m, 5H), 7.10 – 7.03 (m, 2H), 4.48 – 4.28 (m, 2H), 3.78 (dddd, *J* = 12.1, 9.0, 5.6, 2.6 Hz, 1H), 3.66 – 3.54 (m, 1H), 3.49 (t, *J* = 6.9 Hz, 2H), 3.10 (s, 2H), 2.93 (m, 1H), 2.90 – 2.81 (m, 4H), 2.81 – 2.67 (m, 2H), 2.31 (m, 4H), 2.00 (m, 2H), 1.90 – 1.76 (m, 1H), 1.69 (m, 1H); ^{13}C NMR (101 MHz, DMSO) δ 170.68, 169.16, 165.82, 163.80, 158.16, 157.04, 156.31, 155.57, 153.78, 151.74, 143.13, 143.02, 141.25, 137.76, 131.78, 130.74, 130.11, 130.05, 128.60, 128.01, 127.67, 127.31, 126.66, 123.76, 123.51, 118.99, 118.93, 97.32, 91.59, 79.92, 61.18, 57.54, 52.87, 52.80, 52.76, 44.44, 34.90, 31.05, 29.33, 23.93, 15.70; LCMS (*m/z*): 831.3 $[M + H]^+$

4. Chemical structure of JP-2-247 (the positive control).

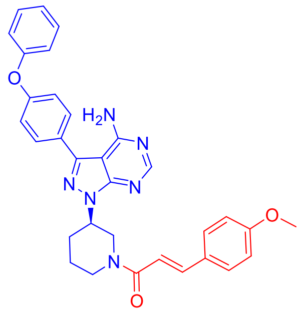

JP-2-247

5. Scanned NMR spectra

^{1}HNMR of 8a in DMSO-D6

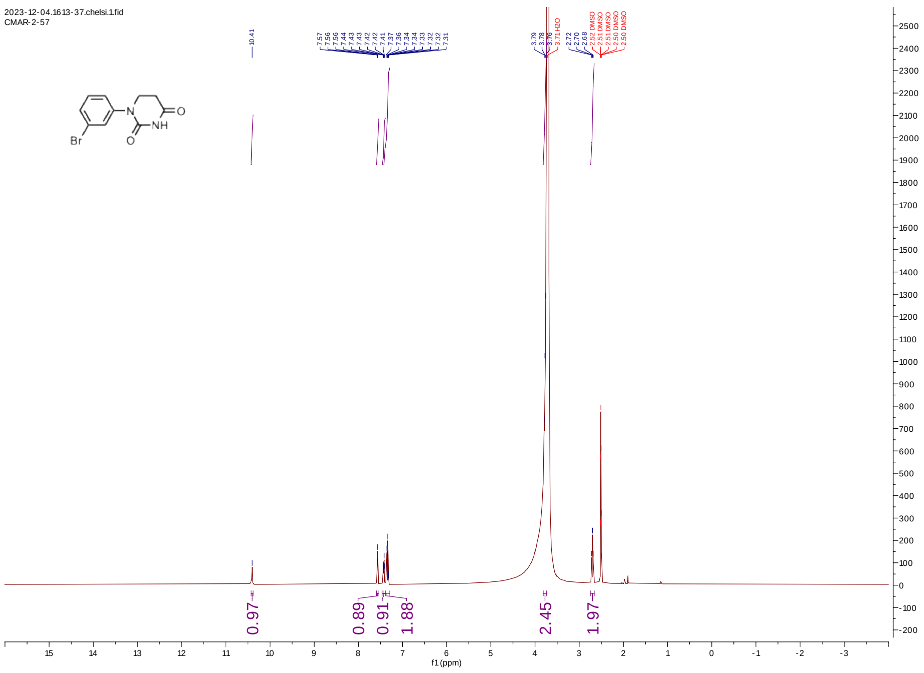

^{13}CNMR of 8a in DMSO-D$_6$

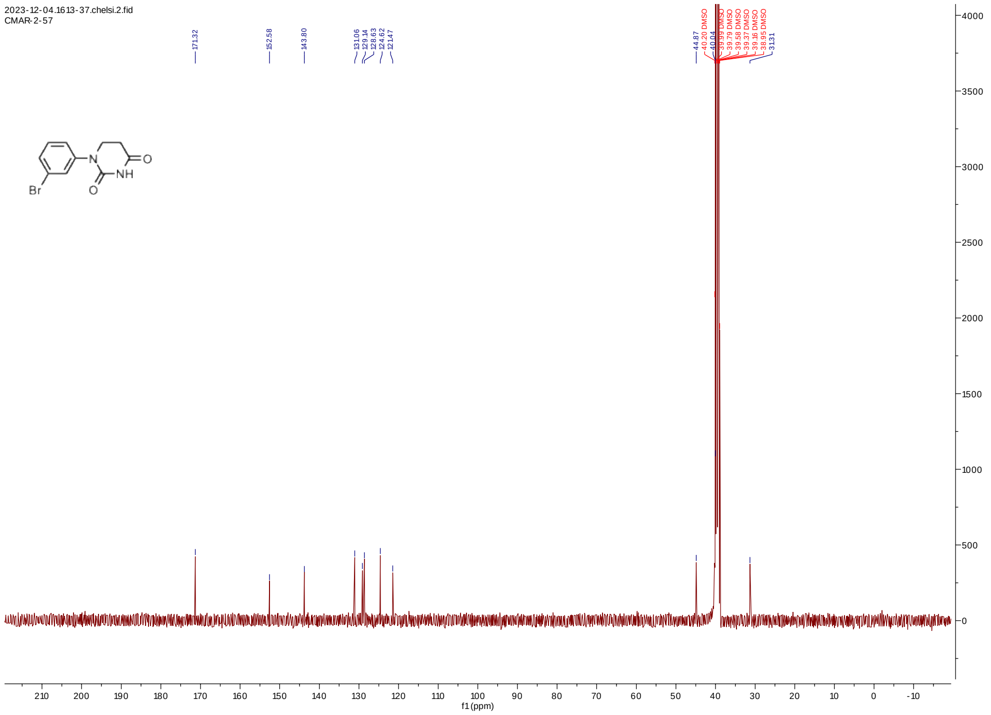

^{1}HNMR of 8b in DMSO-D$_6$

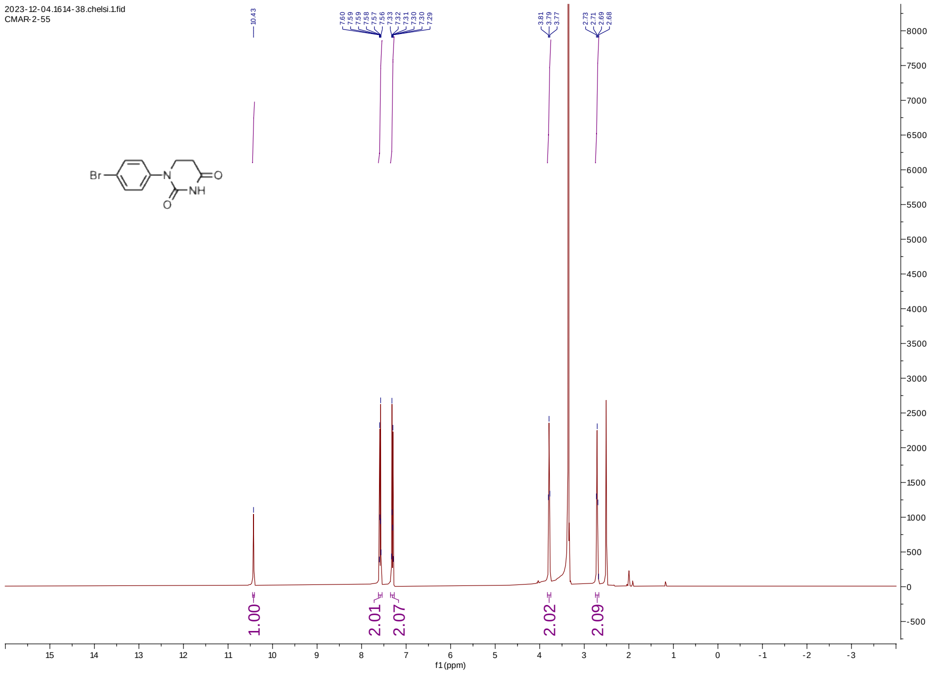

^{13}CNMR of 8b in DMSO-D$_6$

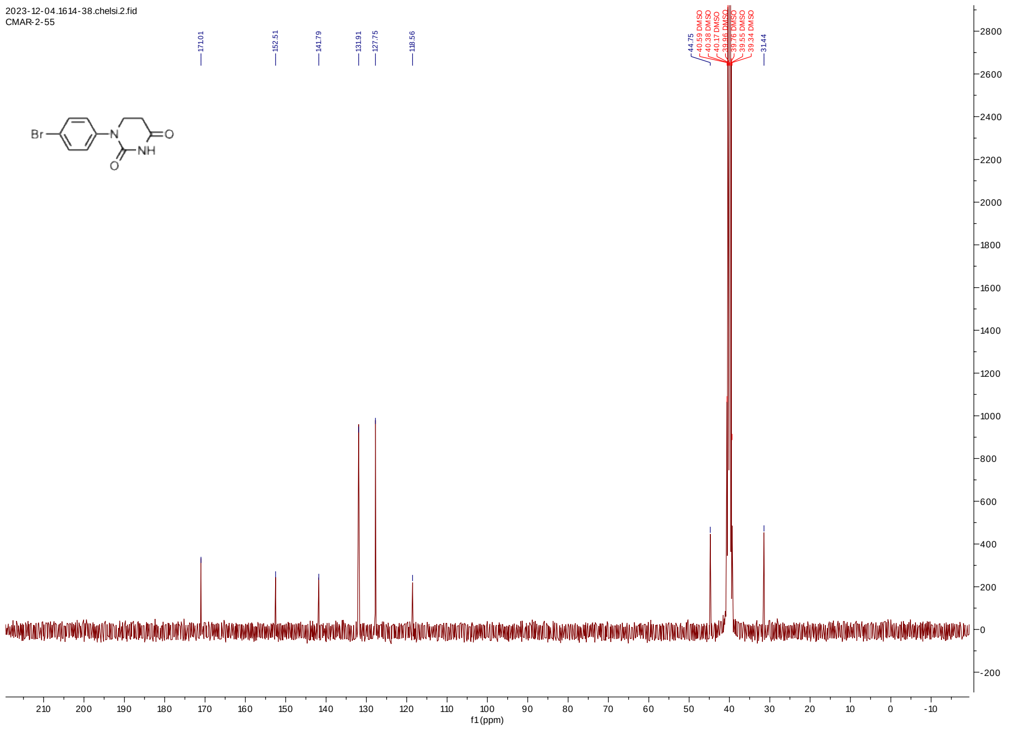

^{1}HNMR of 8c in DMSO-D$_6$

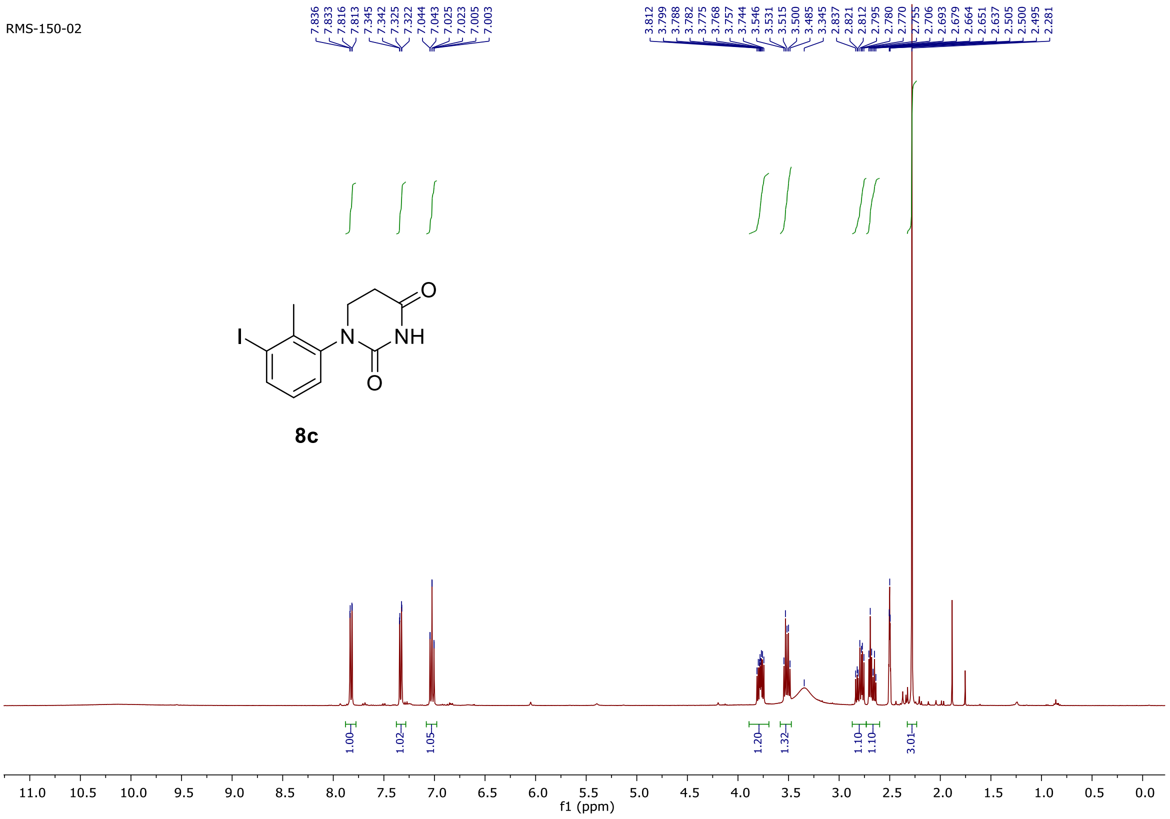

8c

^{1}HNMR of Boc-4 in DMSO-D_6

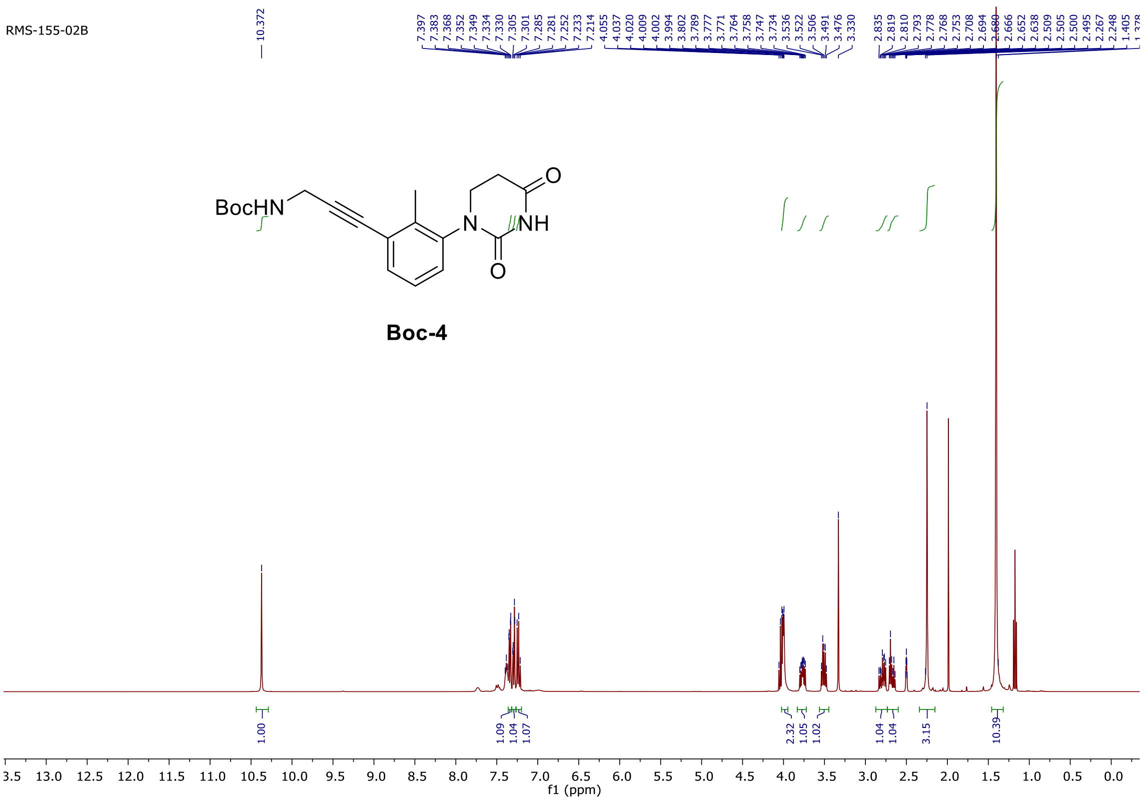

^{1}HNMR of 4 in DMSO-D_6

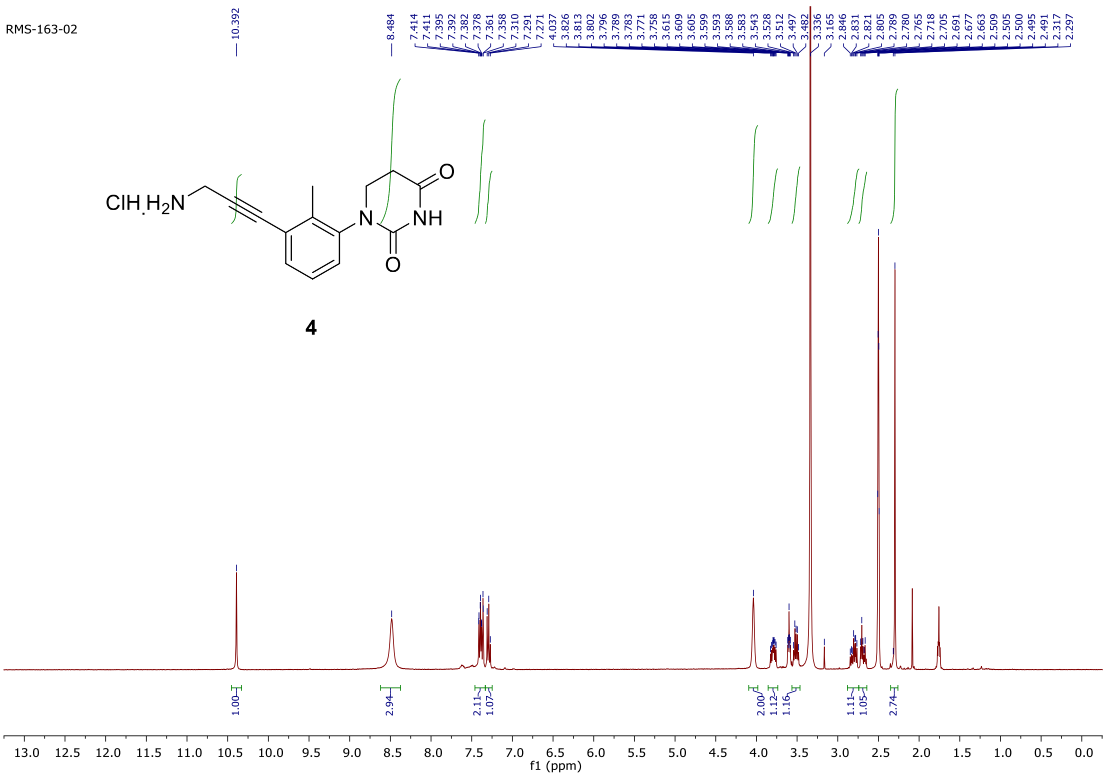

^{1}HNMR of 5a in DMSO-D6

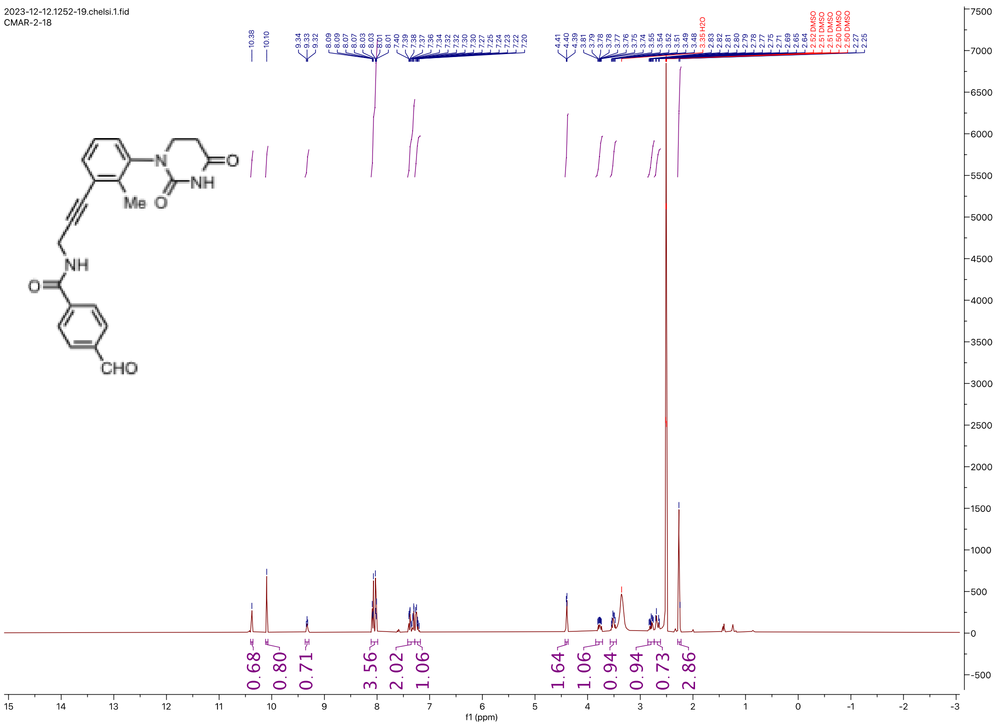

^{1}HNMR of 5b in DMSO-D6

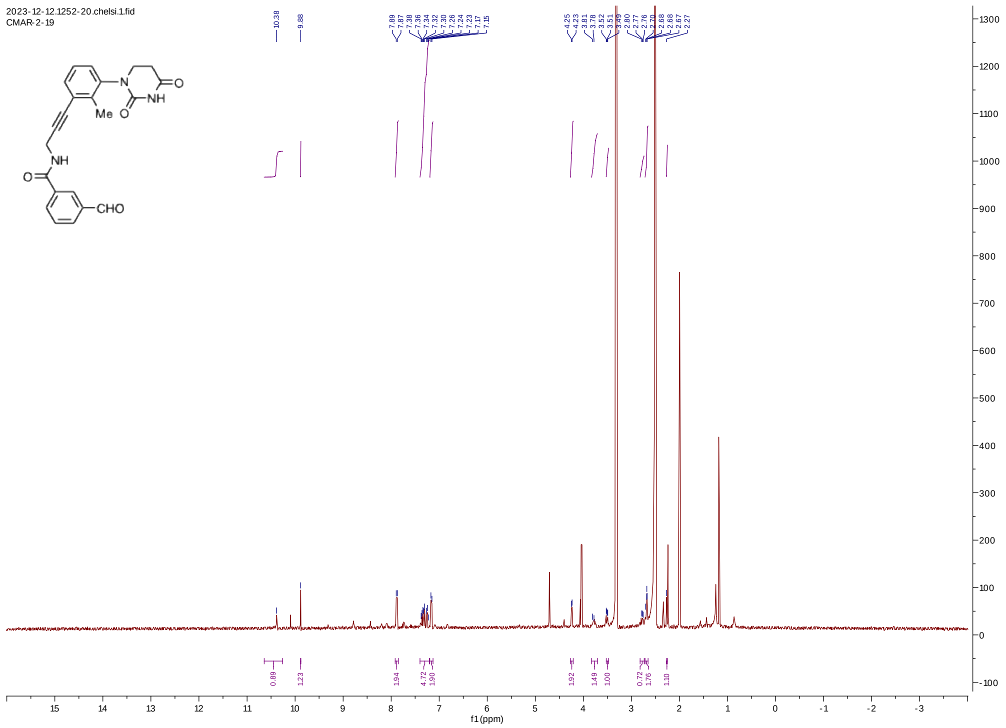

^{1}HNMR of 5c in DMSO-D$_6$

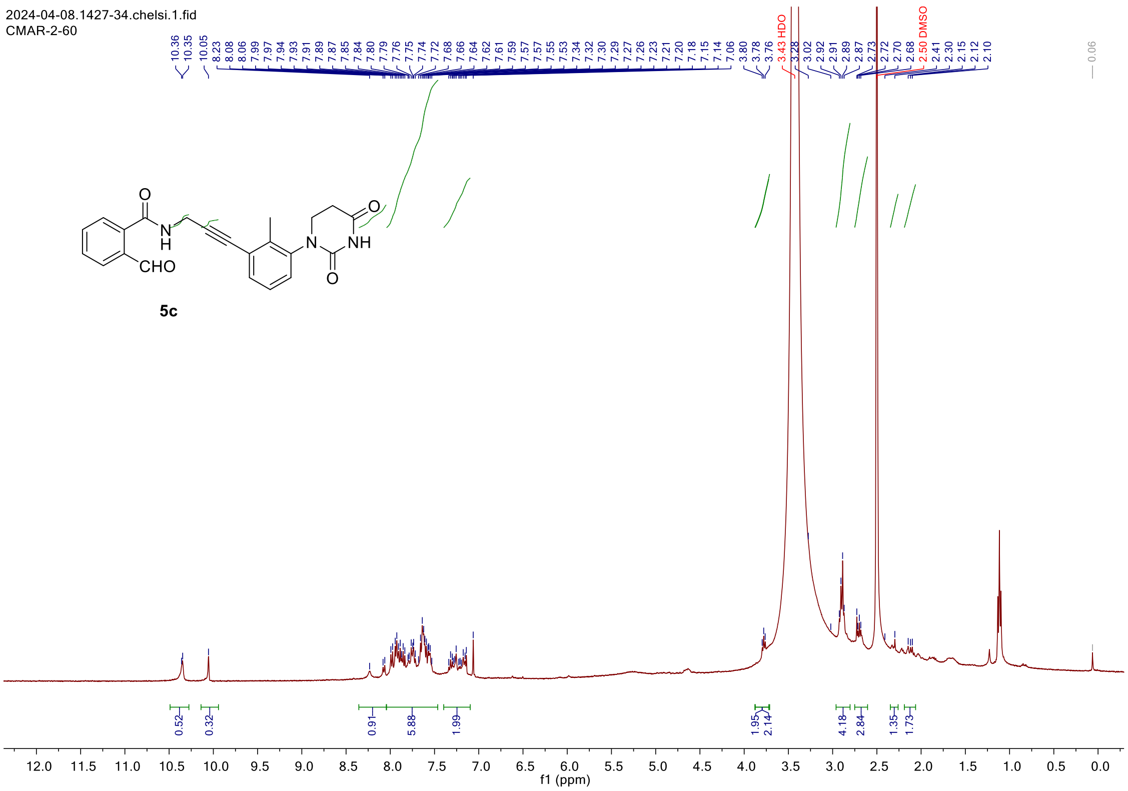

^{1}HNMR of 6a in DMSO-D$_6$

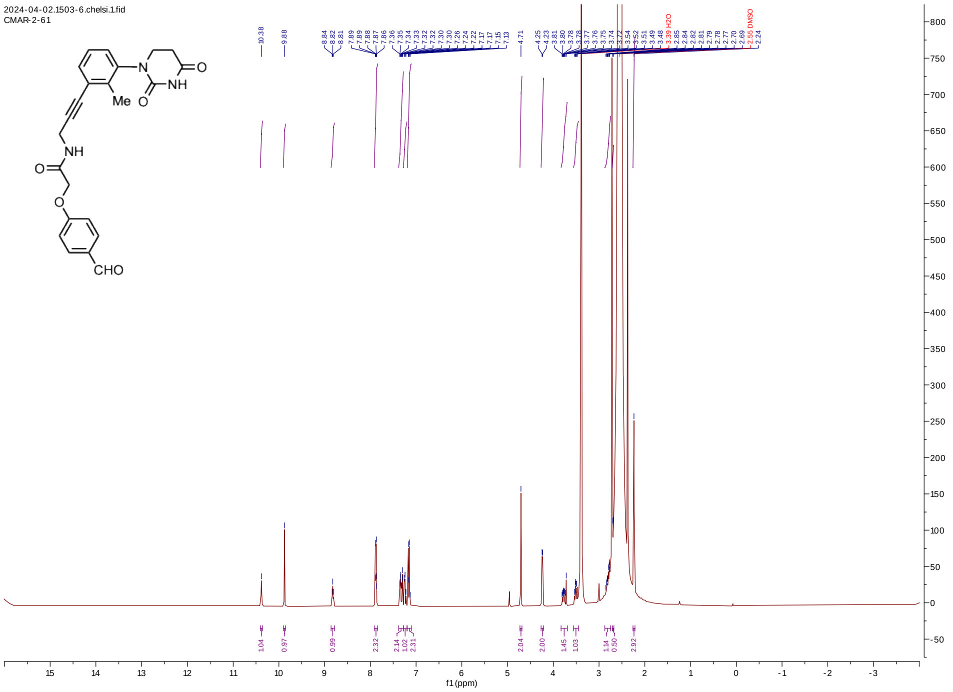

^{13}CNMR of 6a in DMSO-D$_6$

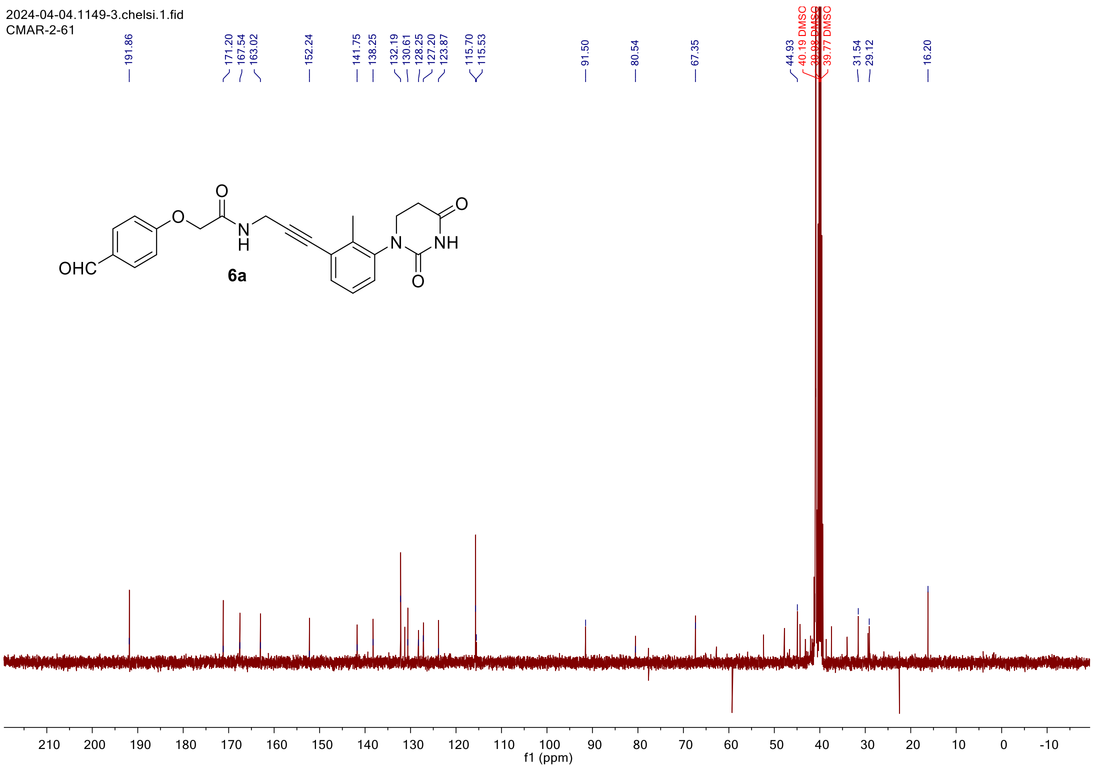

^{1}HNMR of 6b in DMSO-D₆

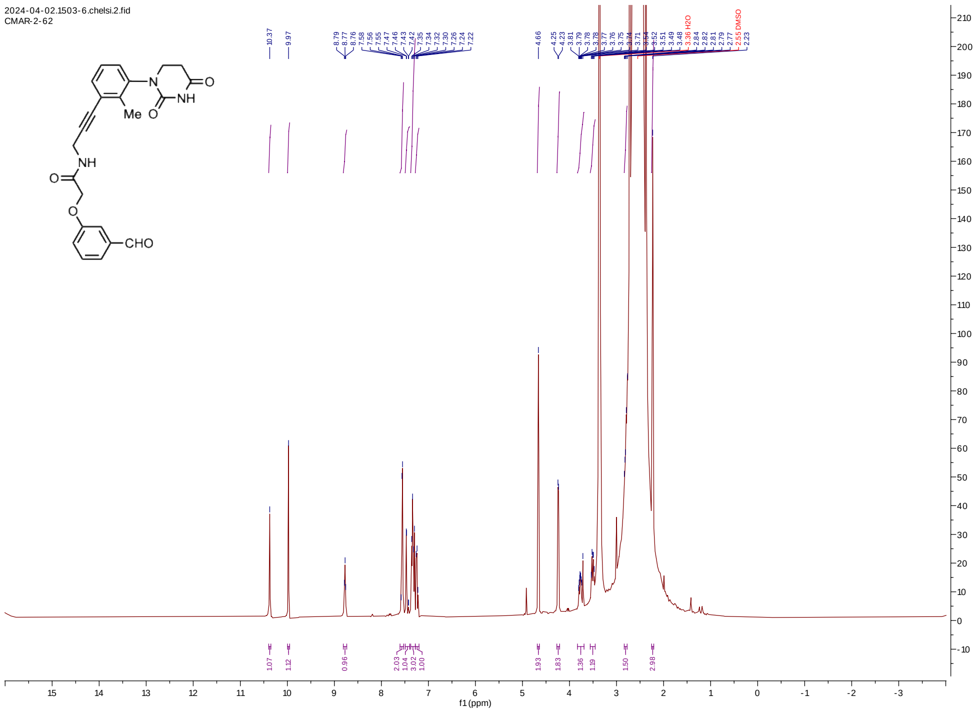

^{13}CNMR of 6b in DMSO-D$_6$

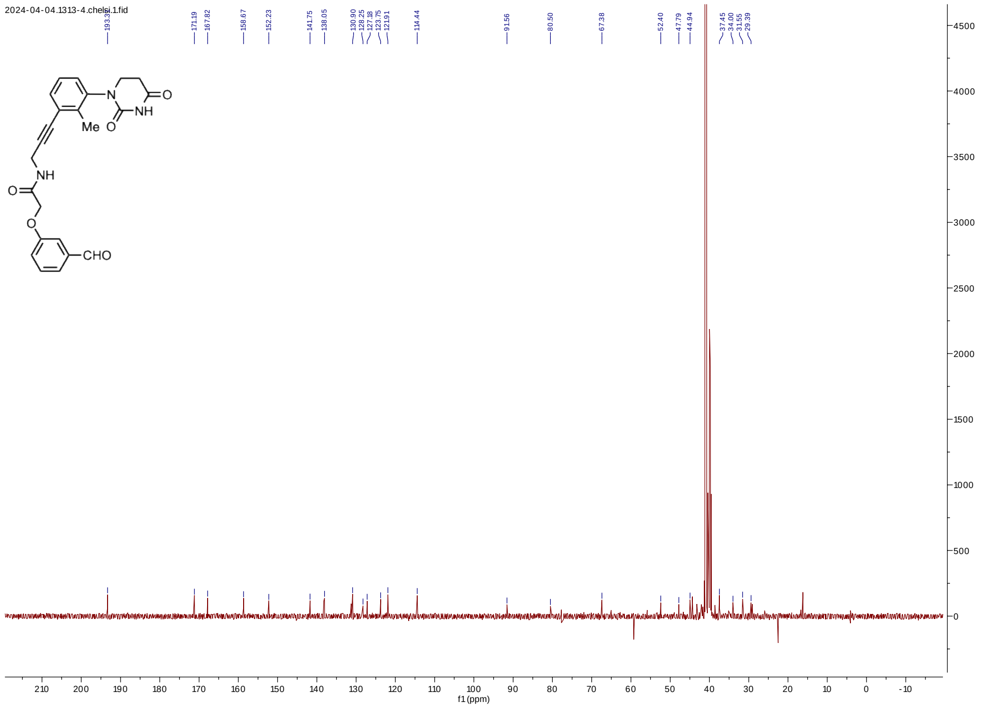

^{1}HNMR of 6c in DMSO-D$_6$

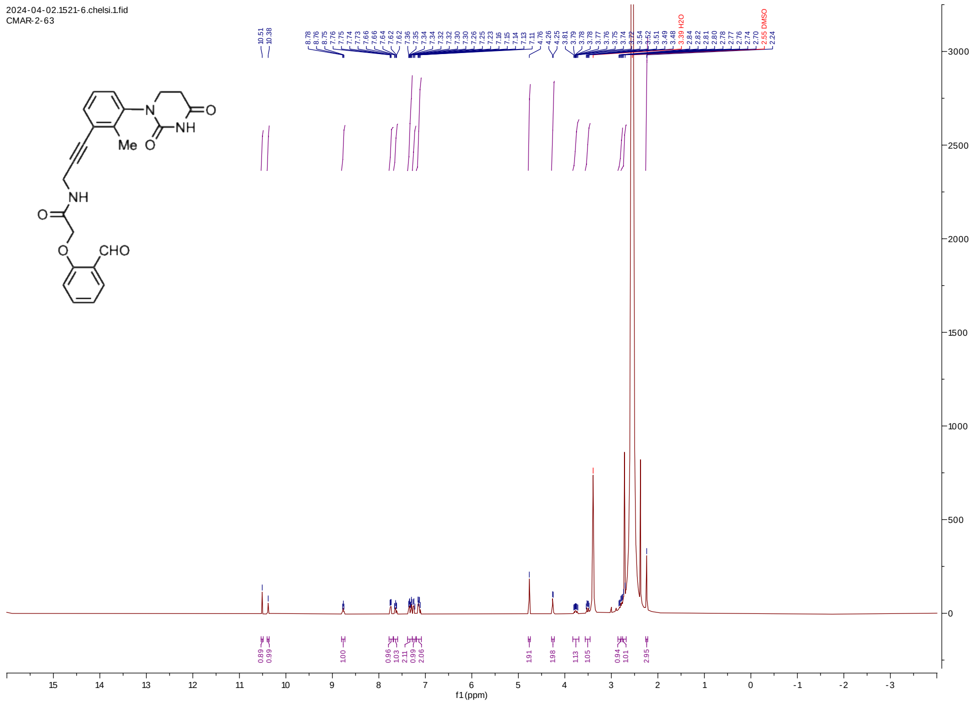

^{13}CNMR of 6c in DMSO-D$_6$

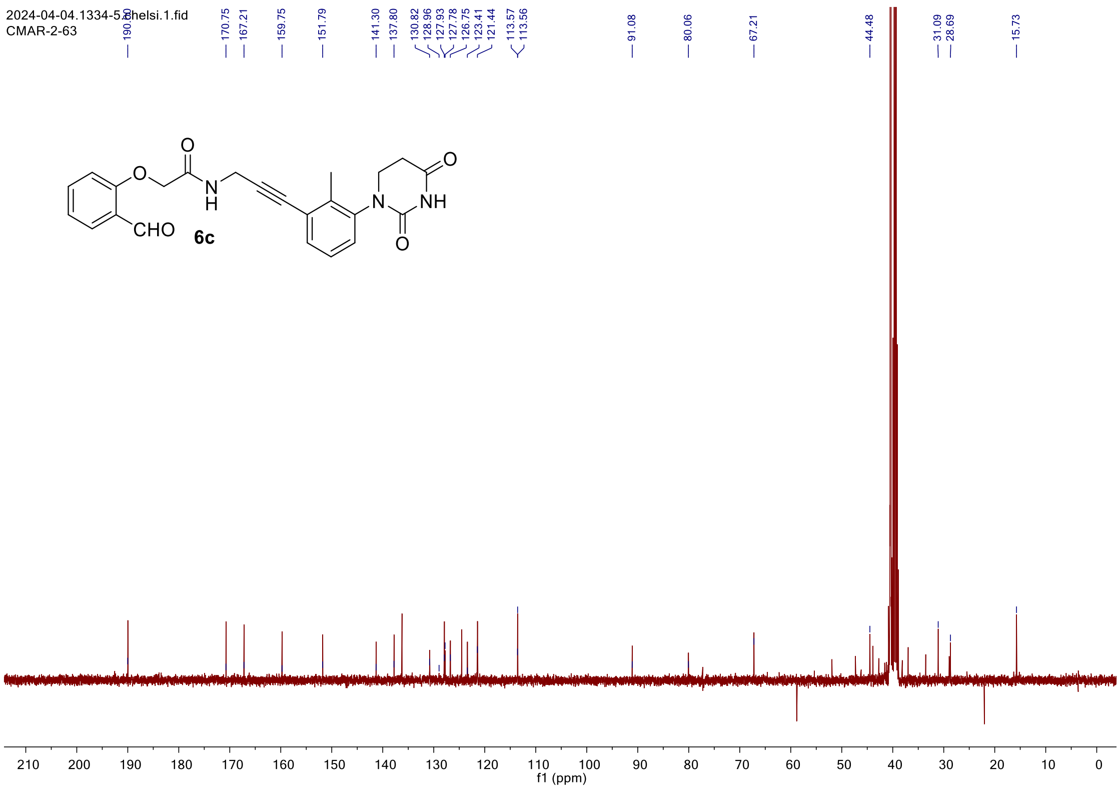

^{1}HNMR of 9a in CDCl$_3$

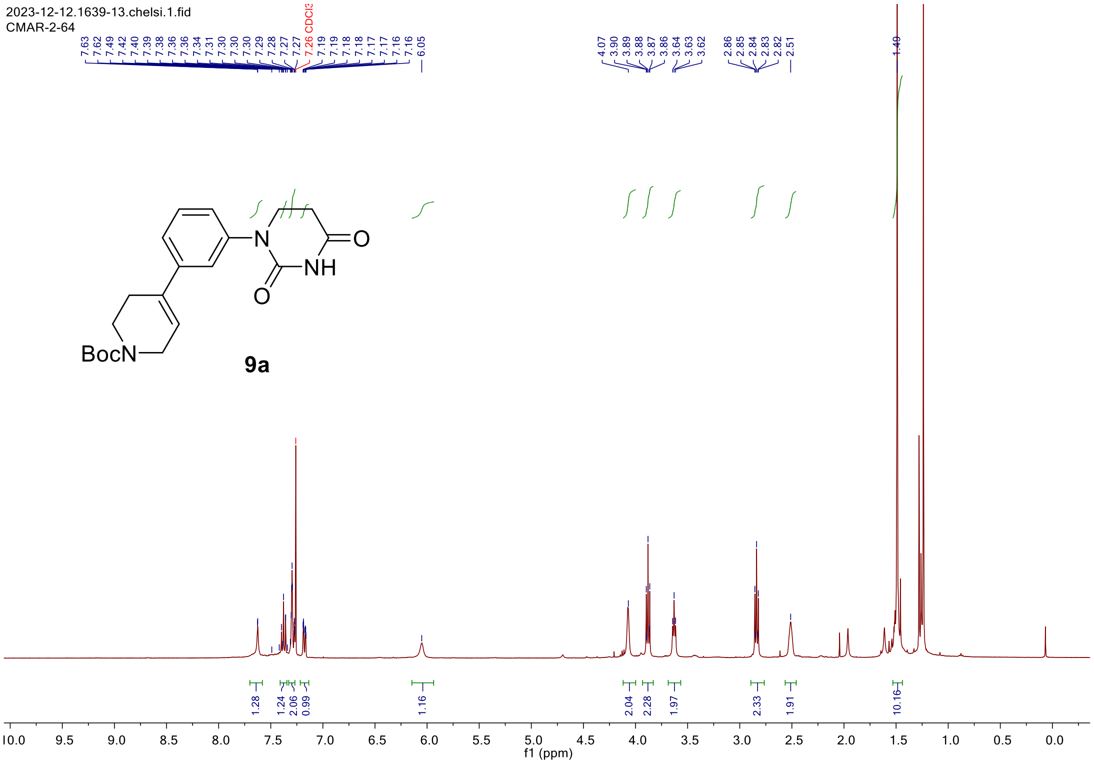

^{13}CNMR of 9a in $CDCl_3$

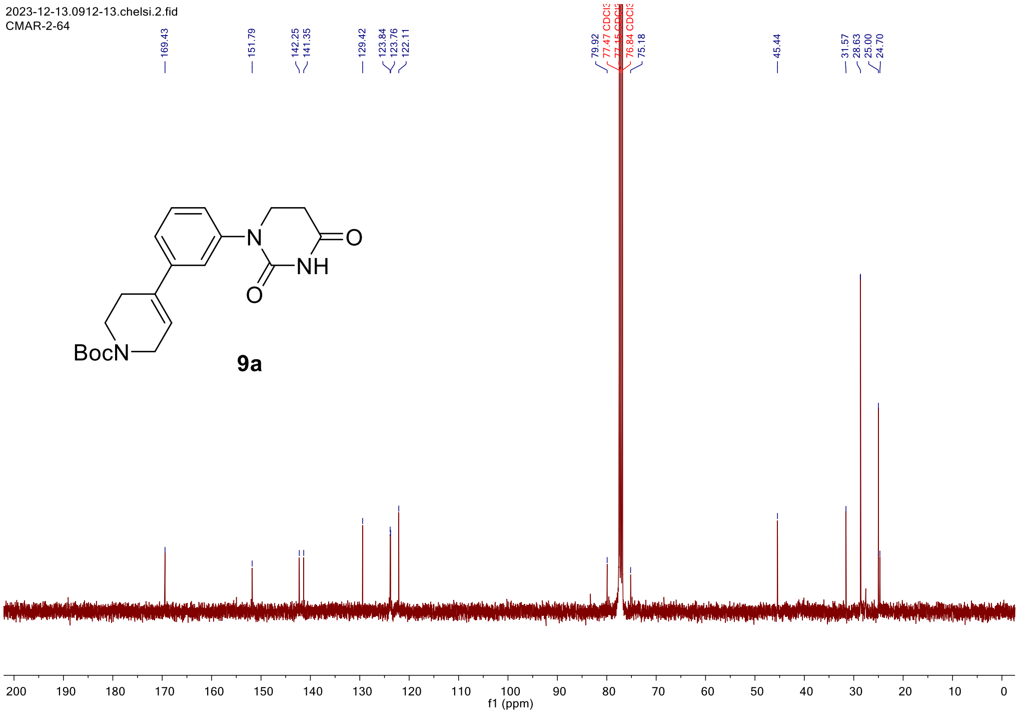

^{1}HNMR of 9b in DMSO-D_6

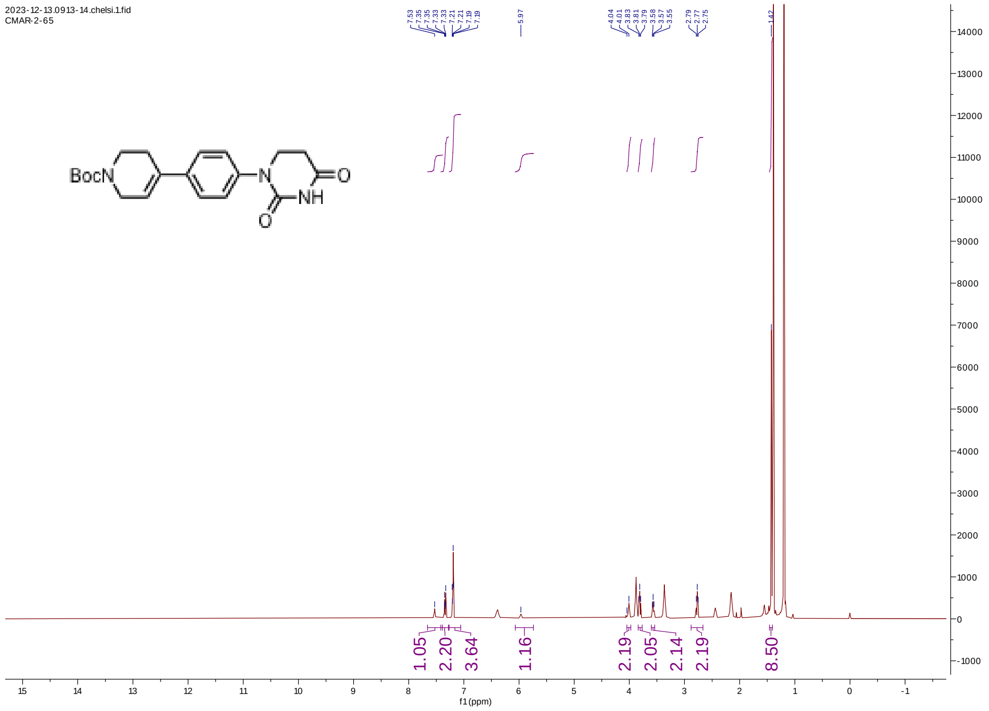

^{13}CNMR of 9b in DMSO-D$_6$

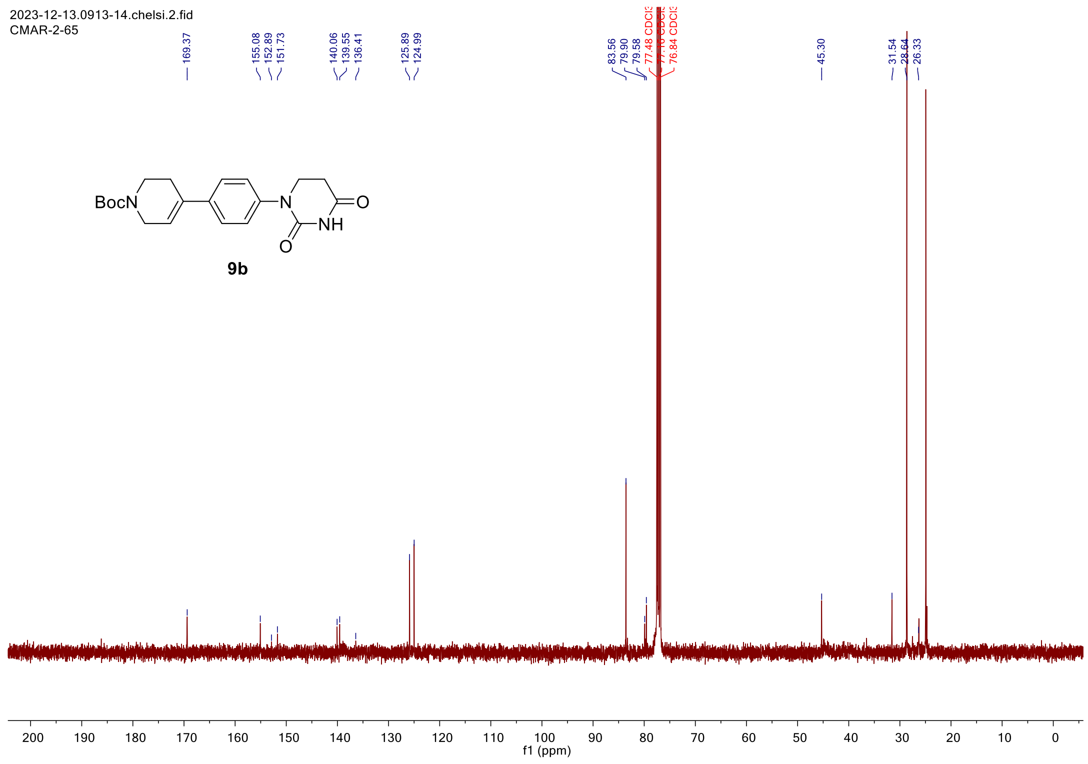

^{1}HNMR of 11aa in DMSO-D$_6$

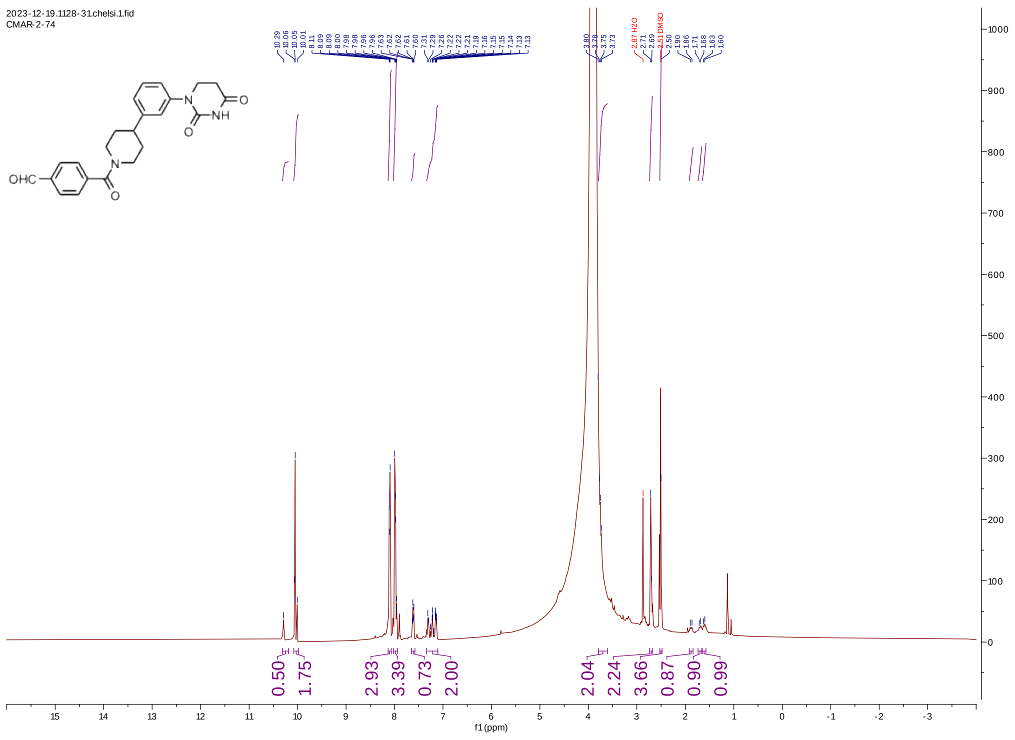

^{1}HNMR of 11ab in DMSO-D_6

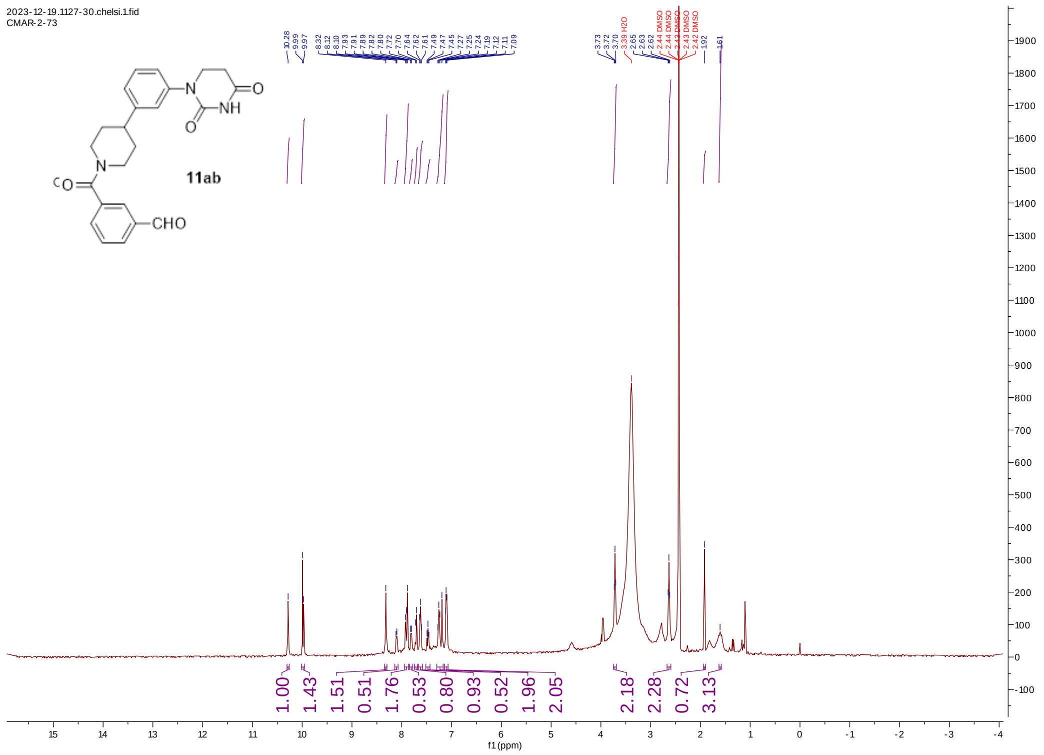

^{1}HNMR of 11ac in DMSO-D_6

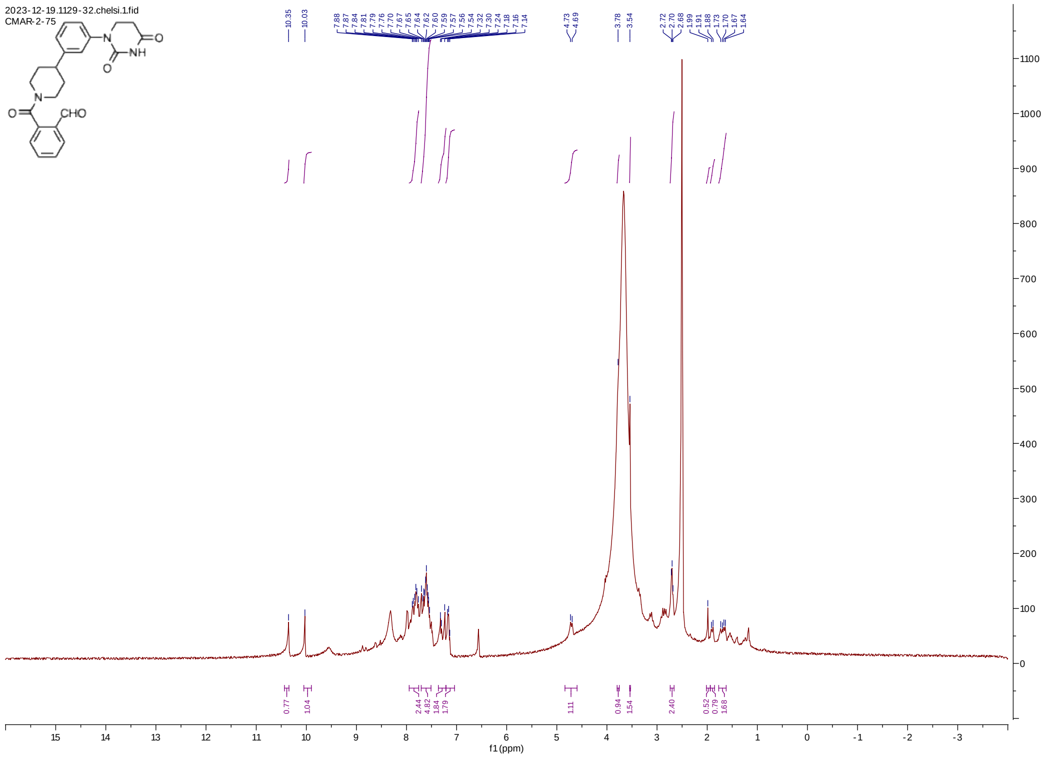

^{11}HNMR of 11ac in DMSO-D_6

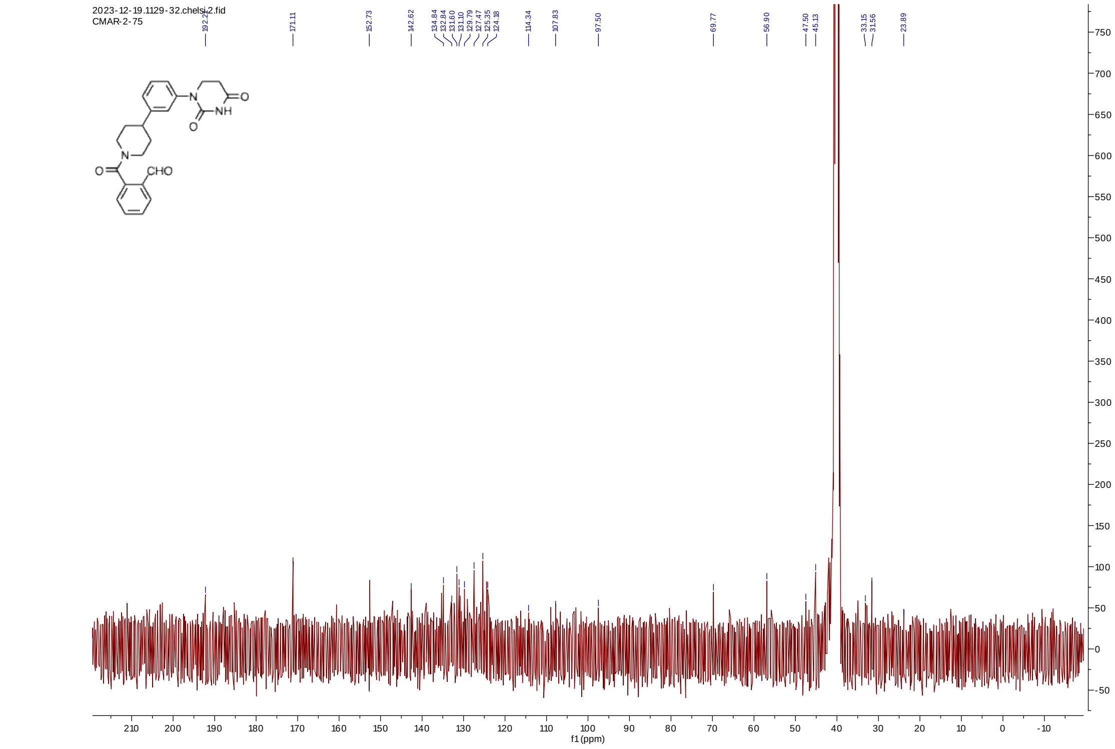

^{1}HNMR of 11ba in DMSO-D_6

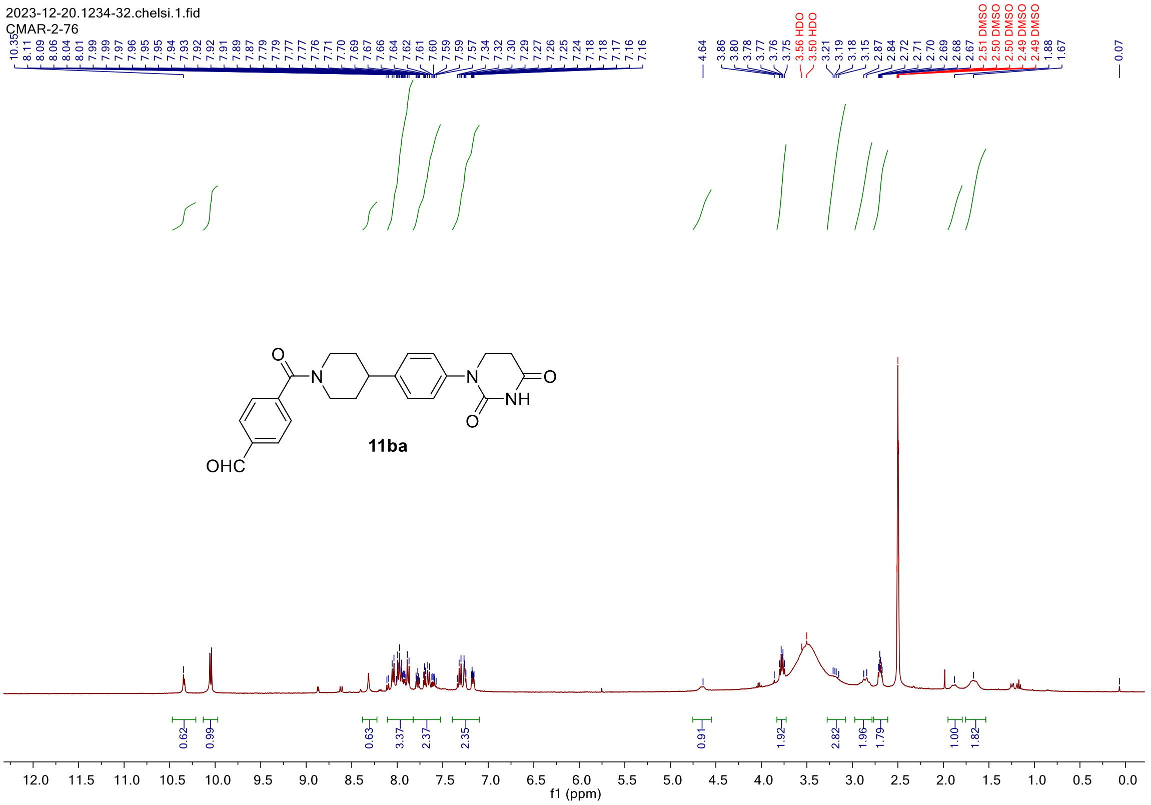

^{1}HNMR of 11bb in DMSO-D$_6$

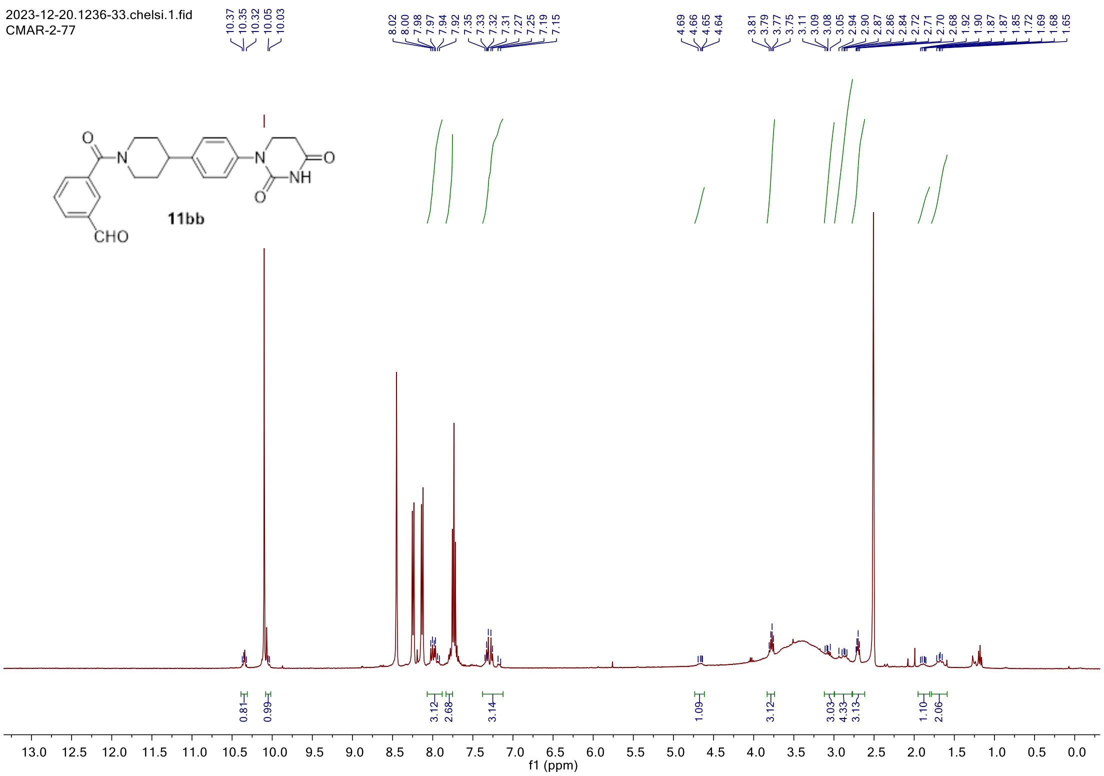

^{1}HNMR of 11bc in DMSO-D$_6$

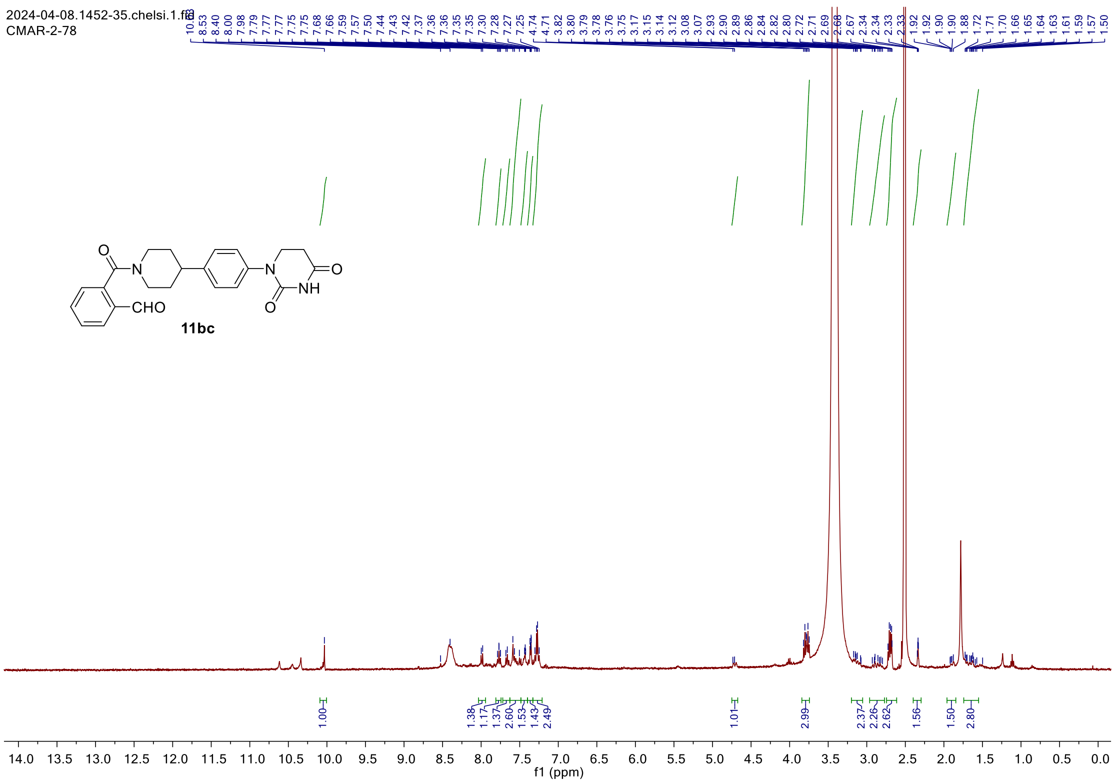

6. BTK Ligands and AM-B1 NMR scanned copies

^{1}HNMR of compound 18 in DMSO-d6

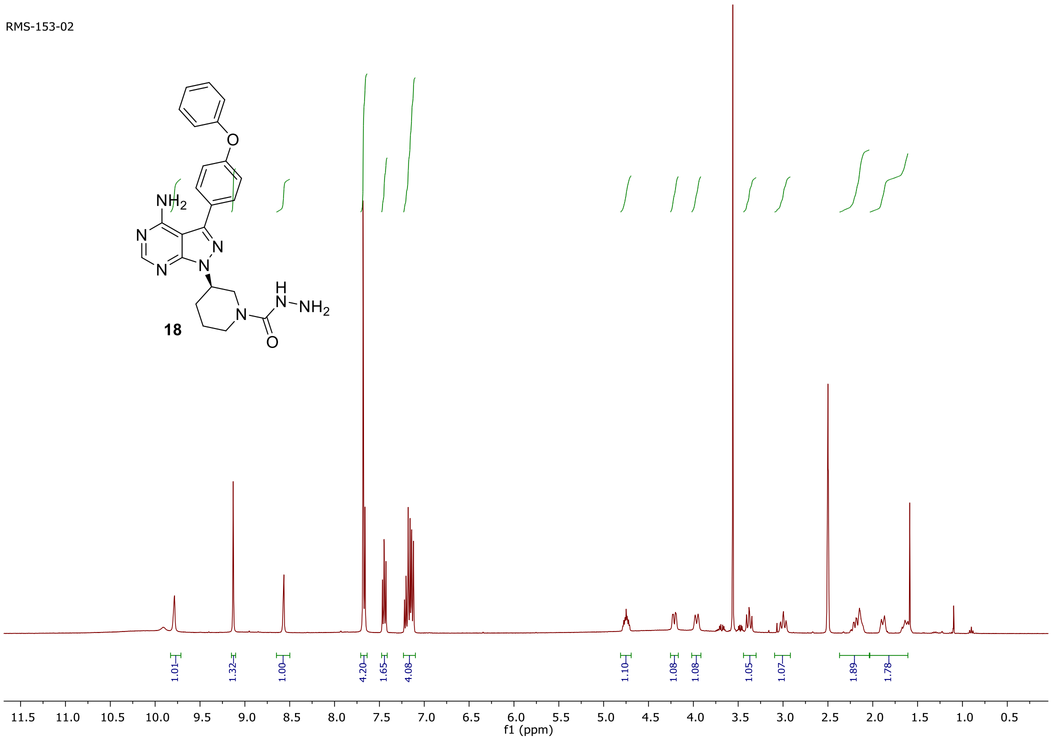

^{13}CNMR of compound 18 in DMSO-d6

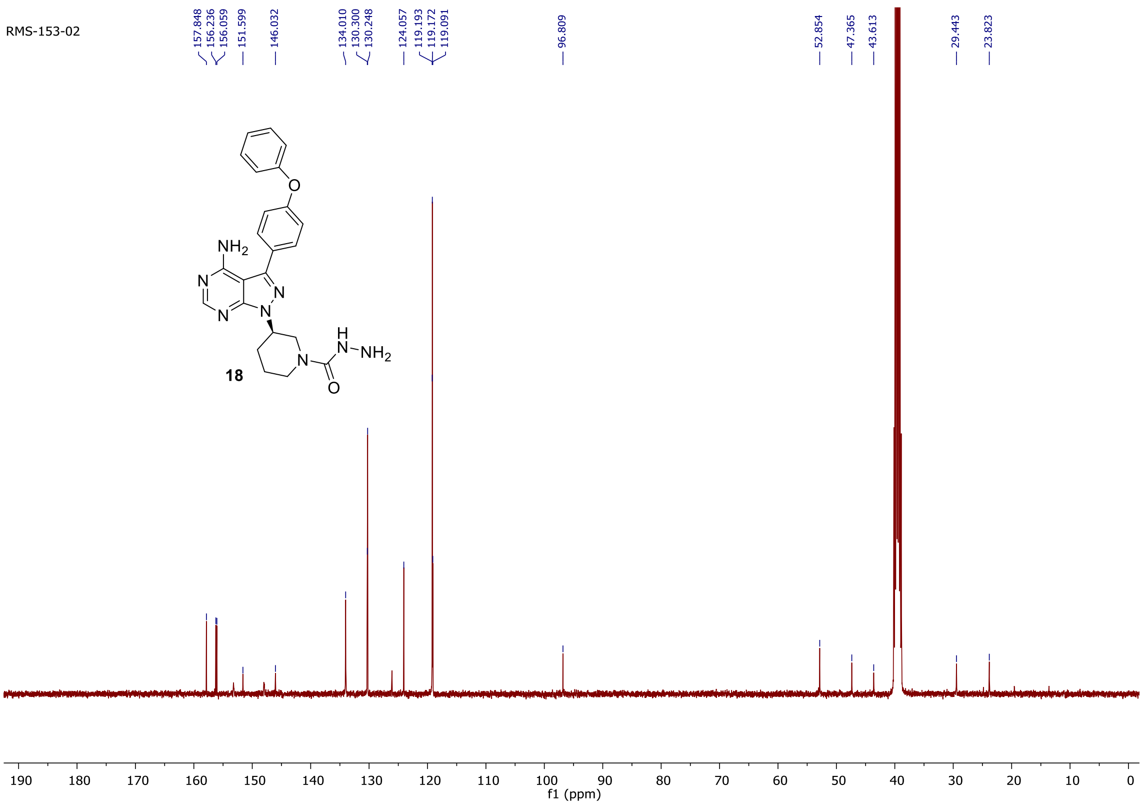

^{1}HNMR of compound 16 in DMSO

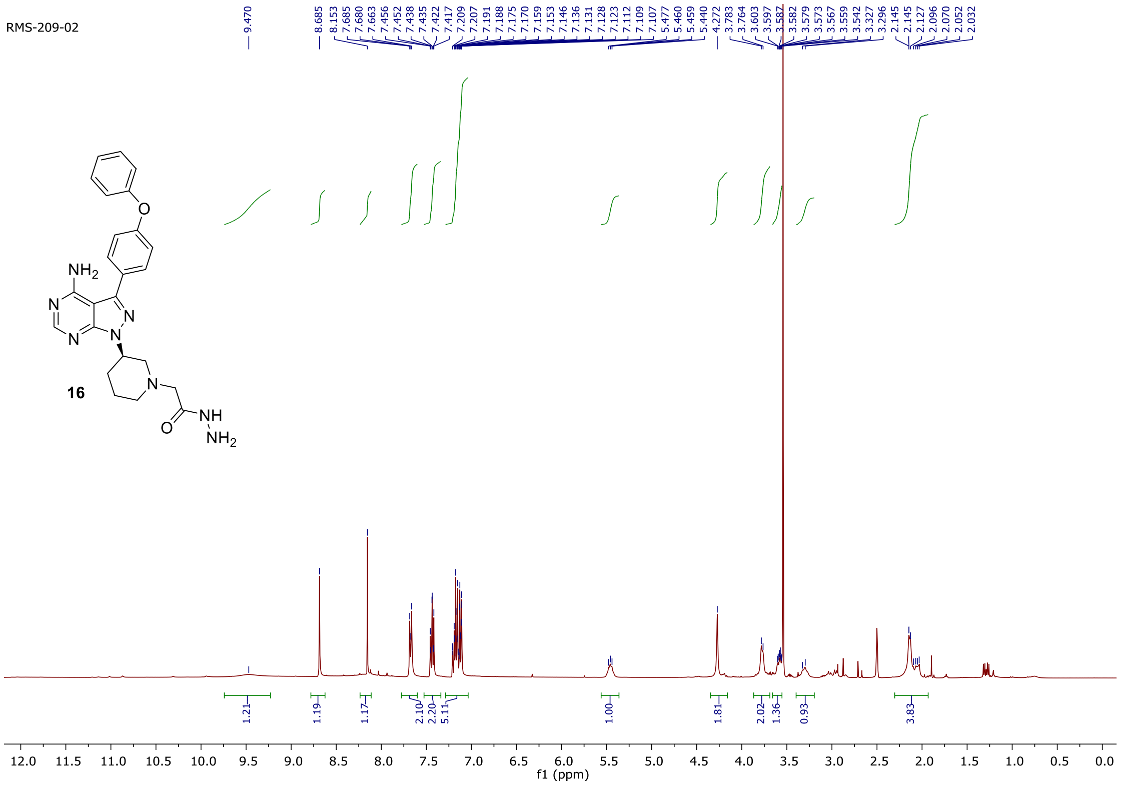

^{13}CNMR of compound 16 in $CDCl_3$

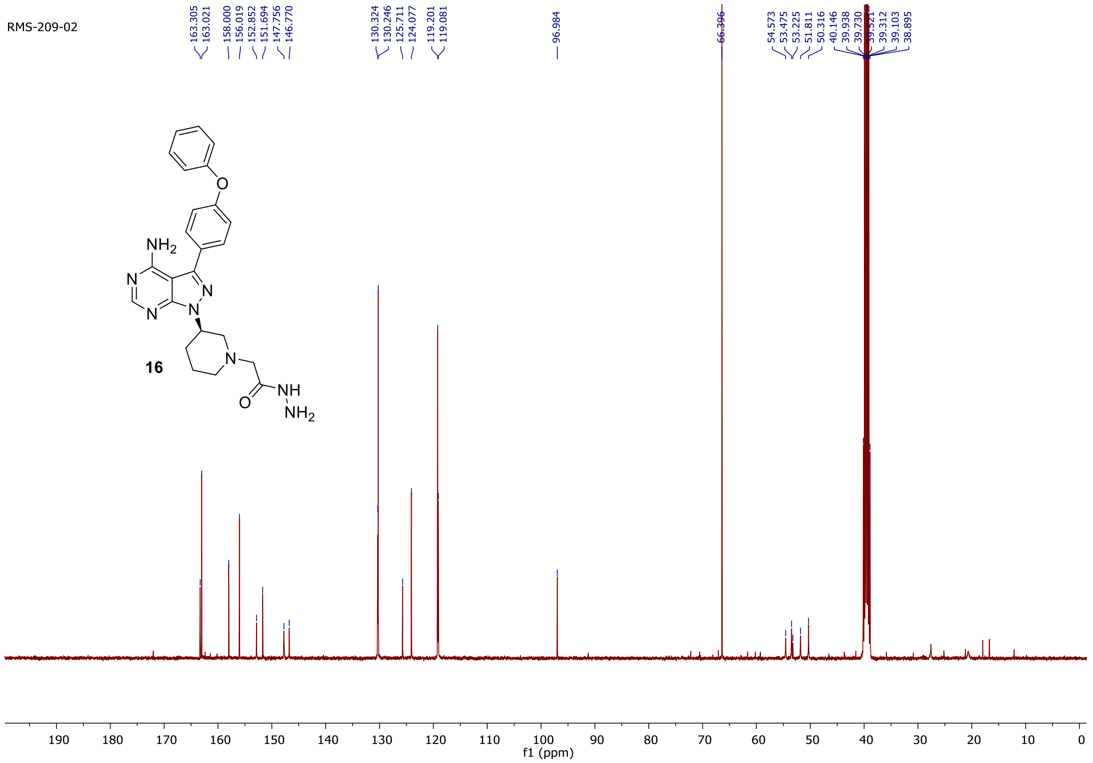

^{1}HNMR of compound 19 in DMSO-D6

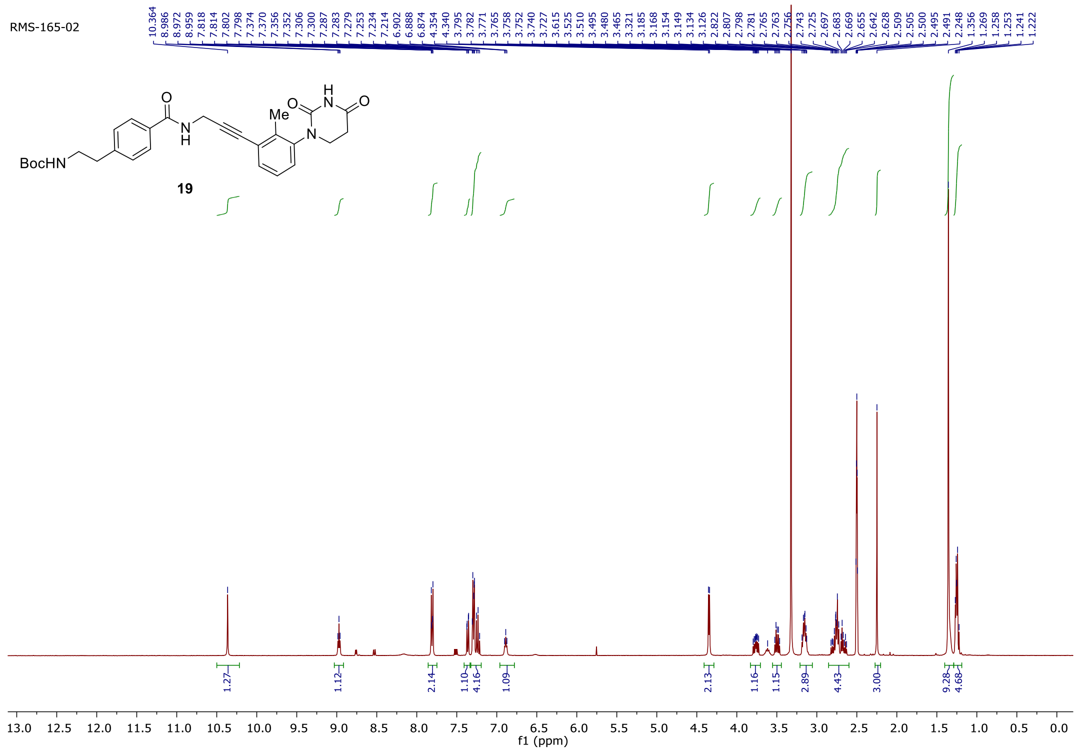

^{1}HNMR of compound 20 in DMSO-D6

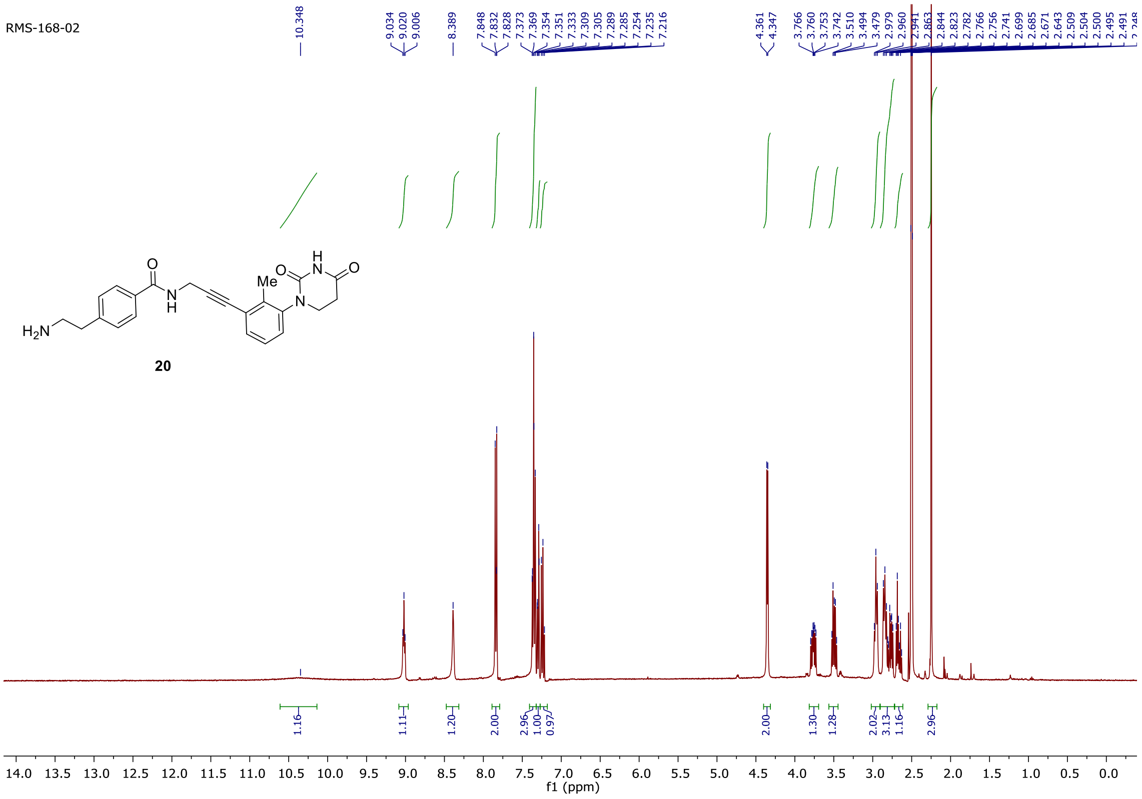

^{1}HNMR of AM-B1 in DMSO-D6

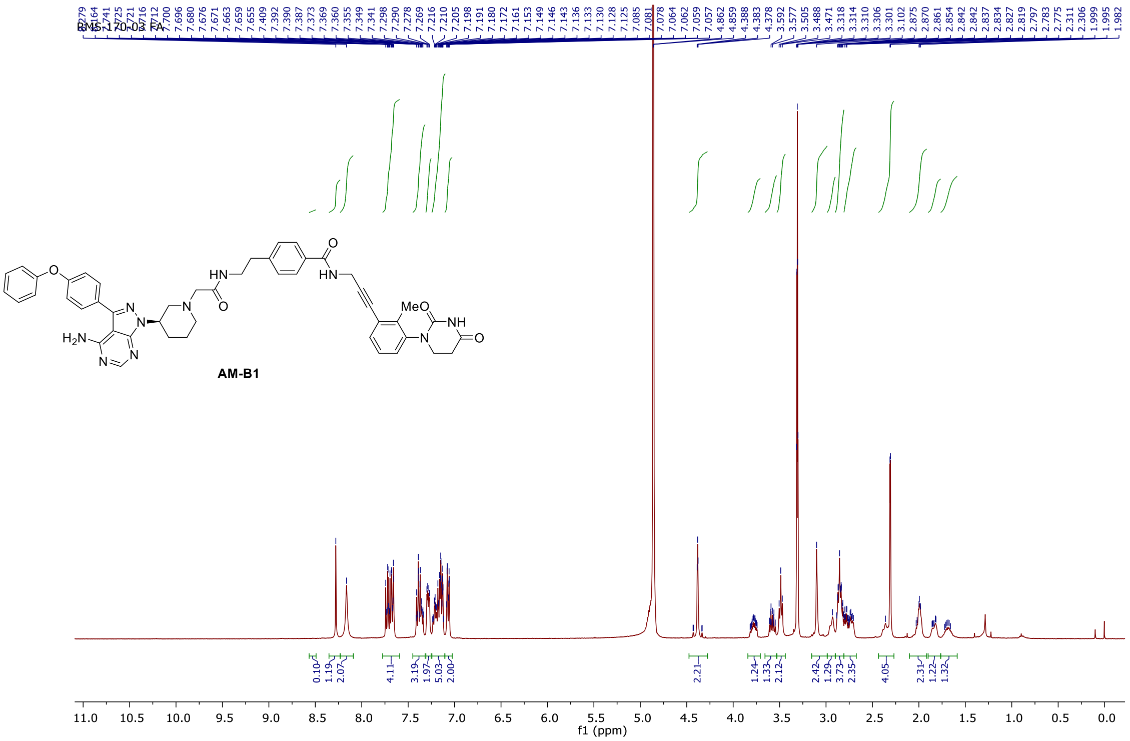

^{13}CNMR of AM-B1 in DMSO-D6

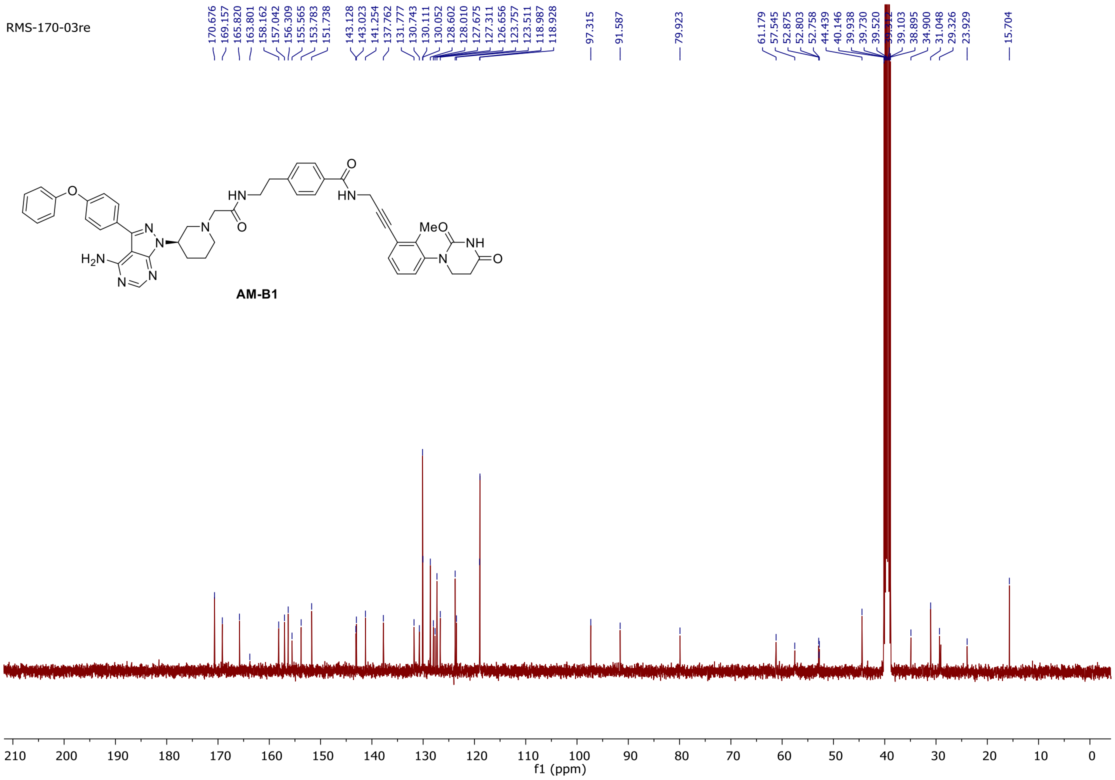

7. HPLC purity of AM-B1

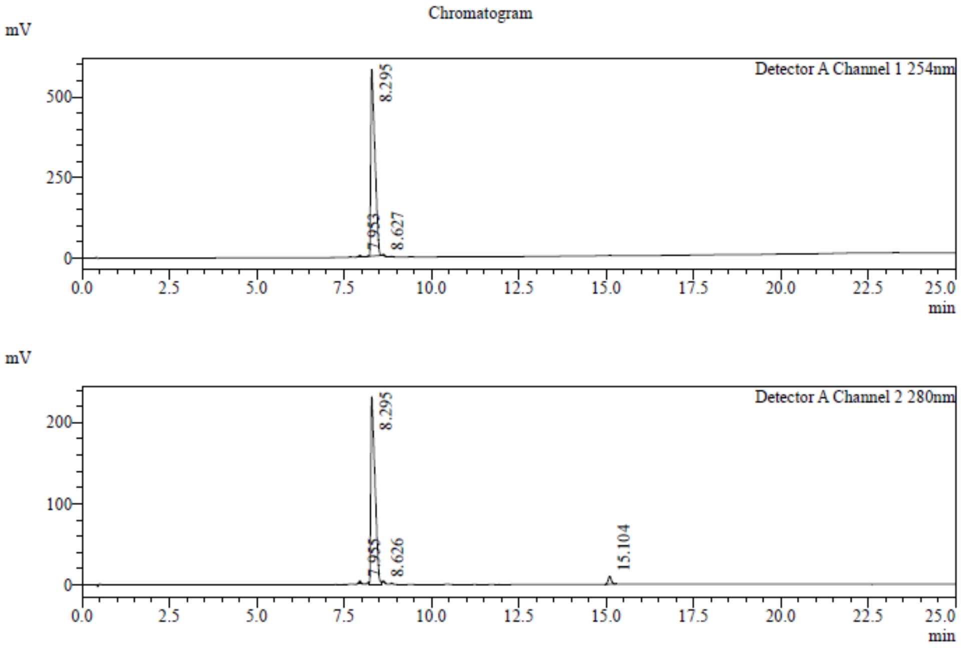

Peak Table

Detector A Channel 1 254nm

Peak#	Ret. Time	Height	Width at 5% Height	Area	Area%
1	7.953	4336	0.109	14728	0.314
2	8.295	576633	0.254	4670360	99.442
3	8.627	3960	--	11472	0.244
Total		584929		4696559	100.000

Detector A Channel 2 280nm

Peak#	Ret. Time	Height	Width at 5% Height	Area	Area%
1	7.955	2955	0.090	8594	0.436
2	8.295	230027	0.259	1901079	96.361
3	8.626	910	--	2355	0.119
4	15.104	9575	0.219	60845	3.084
Total		243468		1972873	100.000

8. DC_{50} of AM-B1 based on the Western blot data in Figure 5A.

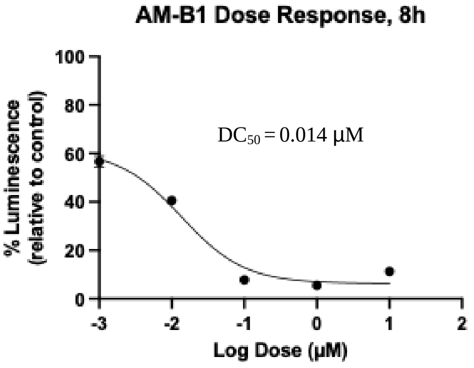

Supplement: Supplementary file 1 — Supplementary Material [file CMDC-20-e202500209-s001.pdf]
